# ROS Self‐Supply Nanoplatform Based on Fenton Catalyst for Chemodynamic and Immunotherapy: Reprogramming Cold Tumor Into Hot Tumor in Cancer Treatment

**DOI:** 10.1002/advs.202523039

**Published:** 2026-04-27

**Authors:** Man Lung Lee, Jack Chun Hin Chen, Wai Wing Cheng, Kwan Yee Lau, Hiu Yee Kwan, Ngar‐Yun Ellen Poon, Hung‐Wing Li

**Affiliations:** ^1^ Department of Chemistry The Chinese University of Hong Kong Hong Kong SAR China; ^2^ School of Biomedical Sciences The Chinese University of Hong Kong Hong Kong SAR China; ^3^ Centre for Cancer and Inflammation Research School of Chinese Medicine Hong Kong Baptist University Hong Kong SAR China; ^4^ Hong Kong Hub of Paediatric Excellence (HK HOPE) The Chinese University of Hong Kong Hong Kong SAR China

**Keywords:** chemodynamic therapy, cold‐to‐hot tumor reprogramming, fenton catalyst, immune checkpoint blockade, immunogenic cell death

## Abstract

The immunosuppressive tumor microenvironment (TME) severely limits the clinical efficacy of immunotherapy, largely due to the prevalence of “cold tumors” with minimal T cell infiltration. To address this, we developed a reactive oxygen species (ROS) self‐supplying nanoplatform (HA‐PGMC) by in situ growing copper peroxide (CuO_2_) nanodots and loading glucose oxidase (GOx) within a mil‐100 metal–organic framework (MOF). HA‐PGMC exploits the catalytic cascade between released Fe^2^^+^ and Cu^+^ ions to generate abundant •OHs (•OH) under acidic and glucose‐rich tumor conditions, thereby amplifying oxidative stress. Meanwhile, Cu^2^^+^ ions deplete glutathione (GSH), further enhancing ROS accumulation. In vitro studies demonstrate that HA‐PGMC induces significant ROS production, GSH depletion, lipid peroxidation, mitochondrial dysfunction, and robust antitumor effects with minimal toxicity to normal cells. Importantly, HA‐PGMC triggers immunogenic cell death (ICD) by promoting CRT exposure and HMGB1 release, effectively converting “cold” tumors into “hot” tumors. This enhanced immune activation paves the way for synergistic combination with immune checkpoint blockade therapy. Collectively, this ROS‐amplifying nanoplatform offers a promising strategy for overcoming the limitations of current cancer immunotherapy by simultaneously inducing potent chemodynamic therapy (CDT) and reprogramming the tumor immune microenvironment.

## Graphical abstract

A multifunctional HA‐conjugated nanoplatform (HA‐PGMC) integrates CuO_2_, glucose oxidase, and mil‐100 to enable cascade catalytic ROS generation in tumor microenvironments. This self‐supplying ROS strategy induces immunogenic cell death, reprograms “cold” tumors into “hot” ones, and synergizes with PD‐L1 blockade, achieving potent chemodynamic–immunotherapy with enhanced tumor inhibition and minimal systemic toxicity.

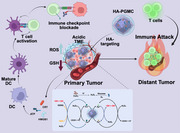

## 1 Introduction

Immunotherapy has brought revolutionary therapeutic benefits for cancer treatment, creating opportunities for cancer patients [1]. In 2011, ipilimumab became the first immune checkpoint inhibitor (ICI) to be approved by the U.S. Food and Drug Administration (FDA), utilizing the CTLA‐4 receptor to realize immunotherapy [2]. On the other hand, the more well‐known Anti‐programmed cell death protein ligand‐1 antibody (αPD‐L1) was approved by FDA in 2016 as an ICI agent to treat cancer [3]. By using immune checkpoint agents, such as αPD‐L1, tumor cells’ immune checkpoint PD‐1 is blocked, restoring the antitumor functions of tumor‐infiltrating lymphocytes (TILs), while side effects are minimized since αPD‐L1 receptors are overexpressed specifically in tumors [4]. However, the response rate in clinical studies remains unsatisfactory, with a mean objective response rate (ORR) of only 19.56% across different cancer types [5]. Therefore, enhancing the efficacy of immunotherapy has become a promising yet challenging task for treating cancers. The major factor contributing to the poor clinical response is the lack of tumor T cell infiltration, characterized as a “cold tumor” — also referred to as immune‐excluded tumors and immune‐desert tumors — where CD8^+^ T lymphocytes only localize at the invasion margin or are absent from tumors and their surroundings [6, 7]. Consequently, increasing the infiltration of T cells prior to applying ICIs is essential for switching a “cold tumor” into a “hot tumor”.

Several strategies have been applied to “heat up” the “cold tumor” into “hot tumor”, including photodynamic therapy (PDT), chemodynamic therapy (CDT), and sonodynamic therapy (SDT) [8, 9, 10]. The underlying mechanism behind these strategies is to induce immunogenic cell death (ICD) by generating excessive reactive oxygen species (ROS) [11]. ICD caused dying tumor cells to release damage‐associated molecular patterns (DAMPs), including calreticulin (CRT), high‐mobility group box 1 (HMGB1), and adenosine triphosphate (ATP), in which DAMPs would be recognized by pattern recognition receptors, prompting the activation of dendritic cells (DCs — also known as antigen‐presenting cells (APCs) [12]. Various PDT, CDT, and SDT agents have been developed for cancer treatment; for example, Qin et al. designed a tumor‐specific, synergistically amplified ICD PDT agent encapsulating ICG to trigger ROS generation by laser irradiation, while Zhan et al. constructed a biomimetic copper‐containing nanogel to generate ROS through Fenton‐like reactions [13, 14]. Nevertheless, despite tumor cells having higher H_2_O_2_ levels (∼100 µm) compared to normal cells, this concentration is still insufficient to induce effective apoptosis via CDT [15]. Moreover, glutathione (GSH) — an antioxidant overexpressed in tumors — serves as a ROS scavenger maintaining oxidative homeostasis and further limiting ROS accumulation. Thus, producing adequate ROS while simultaneously depleting GSH is critical for initiating ICD and enhancing chemodynamic/immunotherapy efficacy [16]. It is therefore vital to develop a ROS self‐supplying nanoplatform capable of both generating large amounts of ROS and reducing ROS consumption within the tumor microenvironment (TME).

Recently, significant efforts have focused on improving the limited H_2_O_2_ levels to enhance CDT efficacy. Among various approaches, glucose oxidase (GOx) has been vigorously utilized to generate H_2_O_2_ by consuming glucose and oxygen. However, this enzymatic process is hampered by the hypoxic tumor microenvironment, significantly limiting its catalytic efficiency [17]. Metal peroxides, composed of metal ions and peroxo groups, have emerged as promising candidates for ROS self‐supplying nanoplatforms, including copper peroxide (CuO_2_), calcium peroxide, and zinc peroxide, which react selectively under acidic conditions to release metal ions and H_2_O_2_ [18]. Various metals — such as Fe^2^^+^, Cu^2^^+^, Mn^2^^+^, and Co^2^^+^ — have demonstrated excellent Fenton catalytic activities, with copper ions particularly exhibiting a potent antitumor effect after being synthesized into copper peroxide [19]. Furthermore, Cu^2^^+^ shows outstanding Fenton‐like catalytic activity under mildly acidic conditions, efficiently converting H_2_O_2_ into highly cytotoxic hydroxyl radicals (•OH), whereas Fe^2^^+^ works optimally at lower pH values (pH 2.0–3.0) [20]. Additionally, Cu^2^^+^ can deplete GSH by converting GSH into GSSG, effectively preventing GSH from scavenging ROS and enhancing tumor oxidative stress [15]. Moreover, Cu derived from CuO_2_ nanodots can catalyze the decomposition of abundant H_2_O_2_ into O_2_, relieving tumor hypoxia [21]. However, despite copper peroxide's remarkable potential in CDT, the synthesized CuO_2_ nanodots are typically 8–10 nm in size, making them prone to rapid renal clearance and poorly suited for tumor accumulation [22].

To date, various ROS self‐supplying platforms have been developed using metal peroxides or metal‐doped Fenton nanozymes, yet each faces distinct limitations. CaO_2_‐based systems generate H_2_O_2_ and Ca^2^^+^ overload but decompose near physiological pH, which compromises tumor selectivity, and they typically require co‐loading of a separate Fe catalyst to drive ·OH generation [23]. MnO_2_‐based platforms excel at O_2_ evolution and GSH depletion, yet their cascade systems commonly rely on additional Fenton components (e.g., FePt, Fe_3_O_4_) for direct ·OH output rather than intrinsic Fenton activity at tumor‐microenvironment pH [24, 25]. Alternative Cu‐based systems — including Cu‐doped ZIF‐8 and CuO_2_ nanodots encapsulated in MOFs — rely on Cu as a dopant or secondary component and often lack a true dual‐metal Fenton cascade [26, 27]. In contrast, the CuO_2_/mil‐100/GOx triad integrates three cooperative mechanisms within a single MOF scaffold: (i) a Cu─Fe dual‐Fenton cascade, in which mil‐100 simultaneously serves as the nucleation template and Fe source, while CuO_2_ provides Cu^2^^+^/Cu^+^ catalytic centers; (ii) pH‐selective, in situ H_2_O_2_ self‐supply via acid‐triggered CuO_2_ decomposition combined with GOx‐mediated glucose‐to‐H_2_O_2_ conversion, which further acidifies the TME and amplifies Fenton kinetics; and (iii) HA‐mediated CD44 targeting coupled with PEG‐PLA shielding to overcome the renal‐clearance limitation of free CuO_2_ nanodots [28]. This integrated design positions HA‐PGMC as a next‐generation ROS self‐supplying platform that couples cascade CDT with ICD‐driven cold‐to‐hot tumor reprogramming for checkpoint blockade synergy [29].

Herein, we designed a ROS self‐supplying nanoplatform (HA‐PGMC) by in situ growing copper peroxide nanodots (CuO_2_) and loading glucose oxidase (GOx) into a metal–organic framework (MOF) to achieve amplified ROS cascade generation for enhanced chemodynamic/immunotherapy. CuO_2_ was grown in situ within mil‐100 — a MOF known for its stable structure, high porosity, and large surface area — serving as an excellent carrier to deliver CuO_2_ into tumors while avoiding renal clearance [30]. Notably, Fe^2^^+^ ions in mil‐100 can also undergo Fenton reactions, catalyzing H_2_O_2_ into •OH upon entry into tumor cells [31]. Furthermore, Cu and Fe ions together establish a catalytic cascade that accelerates the conversion of H_2_O_2_ into ROS. Both CuO_2_ and GOx serve as H_2_O_2_ sources: CuO_2_ decomposes into Cu^2^^+^ and H_2_O_2_ under acidic conditions, while GOx catalyzes the oxidation of glucose into H_2_O_2_, fueling the Fenton and Fenton‐like reactions mediated by Fe^2^^+^ and Cu^2^^+^, respectively, to generate substantial amounts of •OHs. The elevated ROS levels induce ICD and trigger DAMP release, thus stimulating a robust antitumor immune response. Overall, this ROS self‐supplying nanoplatform holds great promise for converting cold tumors into hot tumors and synergizing with ICI immunotherapy to achieve superior cancer treatment outcomes.

## 2 Results and Discussion

### 2.1 Synthesis and Characterization of HA‐PGMC

**SCHEME 1:**
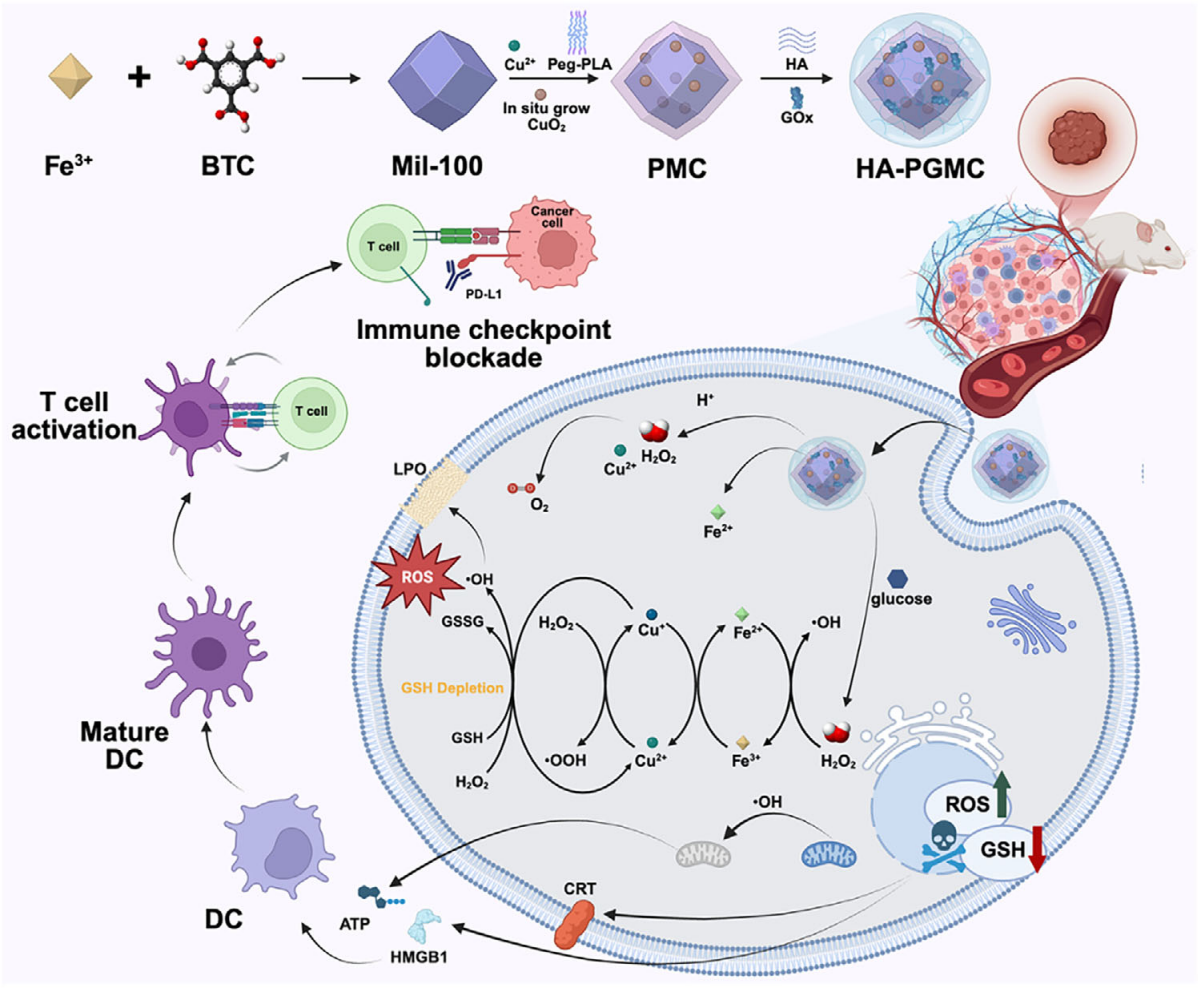
Schematic illustration of ROS self‐supplying nanoplatform.

**FIGURE 1:**
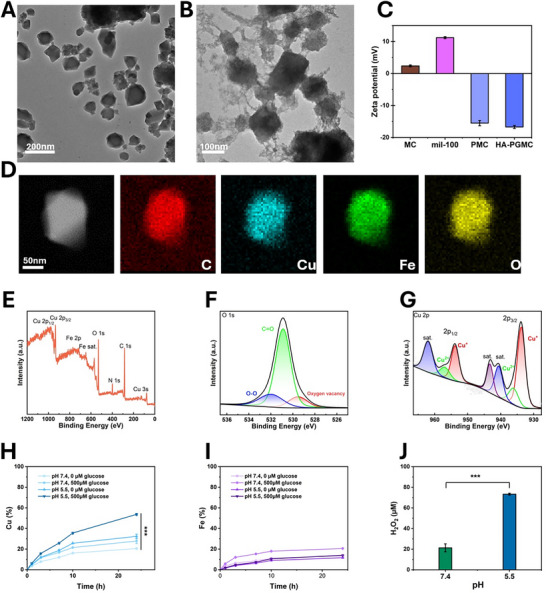
Synthesis and characterization of HA‐PGMC. TEM images of (A) mil‐100 nanoparticles and (B) HA‐PGMC nanoparticles. (C) Zeta potential of MC, mil‐100, PMC, and HA‐PGMC. (D) Corresponding elemental mapping of C, Cu, Fe, and O in HA‐PGMC. (E) Survey XPS spectrum, (F) O 1s high‐resolution XPS spectrum, and (G) Cu 2p high‐resolution XPS spectrum of MC. (H) Cu and (I) Fe ion release profiles of HA‐PGMC under different pH and glucose conditions. (J) H_2_O_2_ generation by MC at pH 7.4 and 5.5. Data are presented as mean ± SD (n = 3). Statistical significance was determined by one‐way ANOVA with Tukey's post‐hoc test (^*^ *p* <0.05, ^**^ *p* <0.01, ^***^ *p* <0.001).

The synthesis of HA‐PGMC is illustrated in Scheme 1. As the carrier of the nanoplatform, the metal–organic framework (MOF, mil‐100) was first synthesized via a microwave‐assisted digestion method by mixing iron(III) chloride hexahydrate with trimesic acid (TMA). Transmission electron microscopy (TEM) revealed that mil‐100 displayed uniform, well‐dispersed hexagonal crystalline nanoparticles (Figure 1A). Dynamic light scattering (DLS) indicated an average particle size of ∼152 nm (Figure S1).

Next, copper peroxide (CuO_2_) was grown in situ on the surface of mil‐100 to form copper peroxide‐loaded MOF (MC). Specifically, Cu^2^^+^ ions were first adsorbed onto mil‐100 through coordination with the unsaturated Fe^3^^+^ sites and trimesate carboxylate groups of the MOF framework, which pre‐concentrated the copper precursors at the MOF surface and provided heterogeneous nucleation sites, while PVP served as a capping agent to restrict particle growth and stabilize the nanodots in the ultrasmall regime. Upon dropwise addition of NaOH, H_2_O_2_ was deprotonated to peroxide anion (O_2_^2^^−^), which rapidly coordinated with the surface‐bound Cu^2^^+^ to form CuO_2_ (Cu^2^^+^–O_2_^2^^−^) nanodots directly on the mil‐100 scaffold (Cu^2^^+^ + H_2_O_2_ + 2OH^−^ → CuO_2_ + 2H_2_O) [19]. This in situ strategy ensured uniform decoration of CuO_2_ on mil‐100 while avoiding free nanoparticle formation in solution, with mil‐100 acting as both a structural template for CuO_2_ nucleation and a source of Fe^2^^+^ for downstream Fenton catalysis [32, 33]. The resulting MC retained a morphology similar to that of mil‐100, with the appearance of nanodot attachments (Figure S2). X‐ray diffraction (XRD) confirmed the successful synthesis of both mil‐100 and MC, with a characteristic diffraction peak observed at approximately 10°, present in both samples (Figure S3), suggesting that the structural integrity of mil‐100 was preserved after CuO_2_ growth.

To improve the hydrophilicity of MC, we synthesized a diblock copolymer, poly(ethylene glycol)‐poly(lactic acid) (PEG–PLA), which enhances colloidal stability and circulation time via PEGylation. PEG–PLA was synthesized via ring‐opening polymerization using Sn(oct)_2_ as a catalyst, yielding PEG_44_–PLA_106_ as the final product (Figure S4). MC was then encapsulated by PEG–PLA using a solvent‐switch method, resulting in a polymer‐coated MC (PMC) with an average hydrodynamic diameter of 333 nm (Figures S5 and S6).

To further amplify reactive oxygen species (ROS) generation and enhance tumor targeting, glucose oxidase (GOx) and hyaluronic acid (HA) were incorporated into the polymer bilayer in a single‐step self‐assembly process, forming the final nanoplatform: HA‐PGMC. TEM analysis confirmed that the morphology of HA‐PGMC resembled that of mil‐100 with an observable polymeric membrane (Figure 1B). DLS measurement revealed a hydrodynamic diameter of 251.0 ± 9.7 nm with a PDI of 0.157 ± 0.050, and the nanoparticles maintained colloidal stability over 7 days (Figure S12). The GOx loading efficiency was determined to be approximately 9% via bicinchoninic acid (BCA) protein assay (Figure S7). Quantitative composition analysis by ICP‐OES and BCA assay confirmed successful co‐loading of all active components, with Cu, Fe, and GOx weight percentages of 15.2%, 17.6%, and 0.8%, respectively (Figure S8). To verify that the assembly process did not compromise enzymatic function, the catalytic activity of GOx was measured before and after encapsulation; the encapsulated enzyme retained approximately 90% of the native GOx activity (Figure S9), confirming that GOx remained functional for downstream H_2_O_2_ generation.

To validate successful construction of the nanoplatform, zeta potential and Fourier‐transform infrared spectroscopy (FTIR) analyses were performed (Figure 1C; Figure S10). The zeta potential values showed a progression from +11.2 mV (mil‐100) to +2.4 mV (MC), −16.5 mV (PMC), and finally −16.7 mV (HA‐PGMC). The FTIR exhibited characteristic peaks at ∼2350 cm^−^^1^ (corresponding to CuO_2_) and ∼1750 cm^−^^1^ (PEG–PLA), both of which were present in HA‐PGMC, confirming the successful incorporation of all components.

Energy‐dispersive X‐ray spectroscopy (EDS) mapping further verified the loading of CuO_2_ into mil‐100, as Fe and Cu signals were co‐localized (Figure 1D). The survey spectrum (Figure 1E) revealed characteristic peaks corresponding to Cu 2p_1_/_2_, Cu 2p_3_/_2_, Fe 2p, Fe satellite, O 1s, N 1s, C 1s, and Cu 3s, confirming the elemental composition of MC. The presence of N 1s suggests polyvinylpyrrolidone (PVP) used to stabilize CuO_2_ during in situ growth. In addition, peaks at 532 eV (O─O) and 530.5 eV (C═O) confirmed the presence of peroxo groups in CuO_2_ (Figure 1F). The Cu 2p peaks at ∼935 and ∼956 eV corresponded to Cu^2^^+^, while those at ∼933 and ∼953 eV were assigned to Cu^+^, indicating a mixed valence state, with Cu^+^ predominating (Figure 1G). For iron, the Fe 2p XPS spectrum revealed peaks at 711 and 721 eV (Fe^2^^+^), as well as 715 and 725 eV (Fe^3^^+^), suggesting a coexistence of +2 and +3 oxidation states (Figure S11).

The stability of the nanoparticles was assessed by monitoring their hydrodynamic size using DLS in PBS (pH 7.4) at 37 °C for 7 days (Figure S12). The results confirmed that HA‐PGMC maintained its morphology and structural integrity under physiological conditions. In addition, a hemolysis assay exposing red blood cells to different formulations showed no detectable hemolysis across all groups, indicating good hemocompatibility and suitability for in vivo use (Figure S13).

We next evaluated the release profiles of Cu^2^^+^, Fe^3^^+^, and H_2_O_2_ under different conditions. Since CuO_2_ decomposes into Cu^2^^+^ and H_2_O_2_ in acidic environments, a time‐dependent release was tested across pH values and glucose concentrations (Figure 1H,I). Cu^2^^+^ release was quantified by ICP–OES, with maximum release (∼53%) observed in pH 5.5 PBS containing 500 µm glucose, compared to 32% (pH 5.5 without glucose), 28% (pH 7.4 with glucose), and 20% (pH 7.4 without glucose). Notably, the pH effect was more pronounced than glucose presence, indicating pH‐responsive Cu^2^^+^ release was dominant over GOx‐mediated H_2_O_2_ generation. Fe^2^^+^ release, however, remained relatively constant across all conditions. TEM imaging confirmed morphological degradation of HA‐PGMC following 24‐h incubation in acidic PBS (Figure S14). Additionally, to validate H_2_O_2_ release from CuO_2_, MC samples were incubated for 1 h in pH 5.5 and 7.4 PBS, revealing significantly higher H_2_O_2_ release at pH 5.5 (Figure 1J).

In summary, the HA‐PGMC nanoplatform was successfully synthesized and comprehensively characterized. The CuO_2_ nanodots were efficiently grown within mil‐100, which was subsequently wrapped with PEG–PLA and loaded with GOx and HA. The system exhibited pH‐ and glucose‐responsive Cu^2^^+^ and H_2_O_2_ release, confirming that the catalytic properties of CuO_2_ were well preserved and suggesting strong potential for enhanced CDT against tumors.

### 2.2 Evaluation of Different Functionalities of HA‐PGMC

**FIGURE 2:**
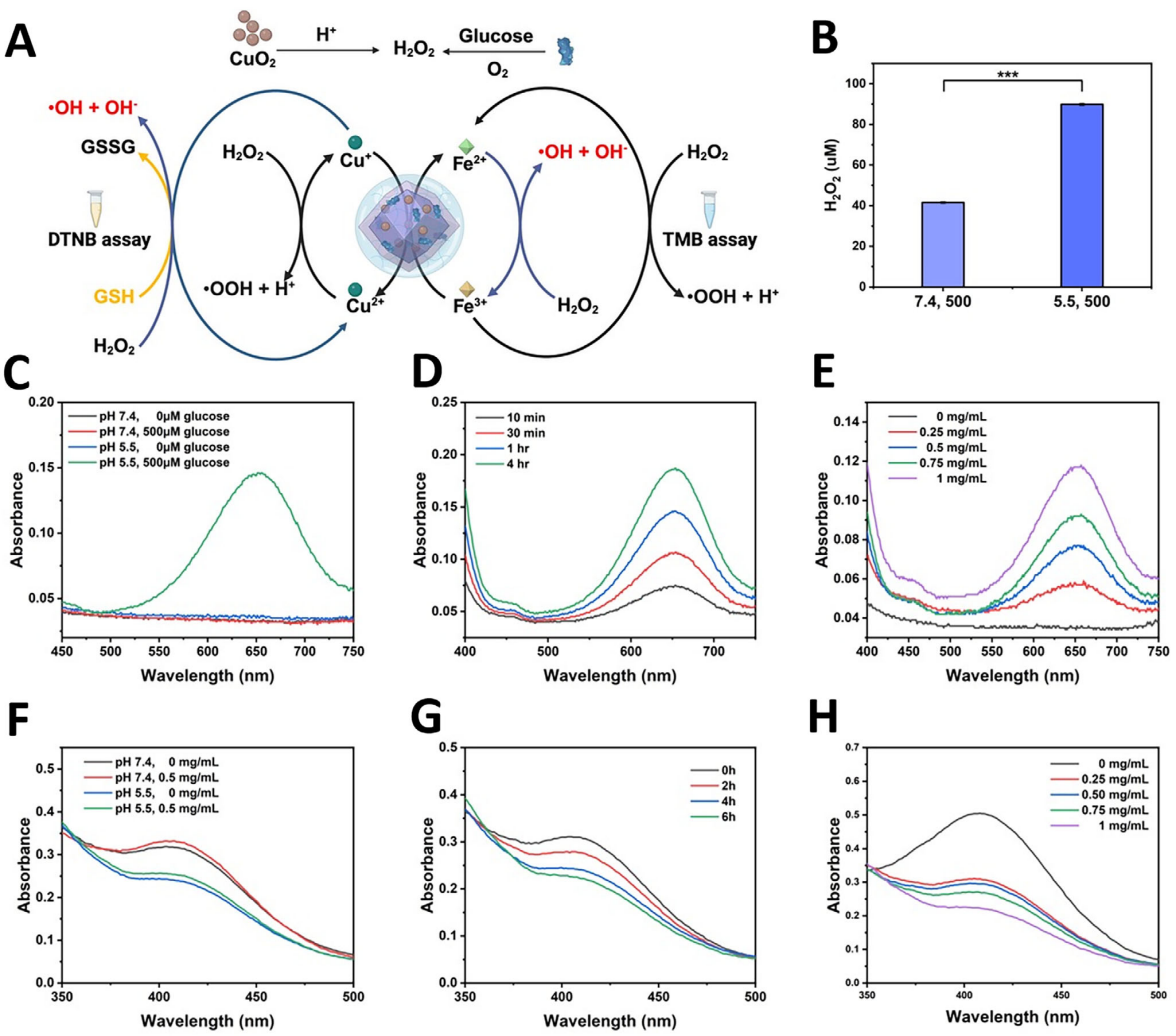
Functional evaluation of HA‐PGMC. (A) Schematic illustration of the Cu‐Fe catalytic cascade and GSH depletion mechanism. (B) H_2_O_2_ generation by HA‐PGMC under different pH and glucose conditions (pH 7.4 and pH 5.5, 500 µm glucose). (C) TMB UV–vis absorption spectra of •OH generation under different conditions. (D) Time‐dependent •OH generation by HA‐PGMC. (E) Concentration‐dependent •OH generation by HA‐PGMC. (F) DTNB UV–vis absorption for GSH depletion under different conditions. (G) Time‐dependent GSH depletion by HA‐PGMC. (H) Concentration‐dependent GSH depletion by HA‐PGMC. Data are presented as mean ± SD (n = 3). Statistical significance was determined by one‐way ANOVA with Tukey's post‐hoc test (^*^ *p* <0.05, ^**^ *p* <0.01, ^***^ *p* <0.001).

The major functionalities of HA‐PGMC are illustrated in Figure 2A, including the Cu–Fe catalytic loop for Fenton and Fenton‐like reactions to generate reactive oxygen species (ROS), production of H_2_O_2_ by GOx, acidic decomposition of CuO_2_, and depletion of GSH via conversion of Cu^+^ to Cu^2^^+^. We first examined the oxygen generation capability of HA‐PGMC by immersing the nanoparticles in acidic PBS (pH 5.5). HA‐PGMC successfully produced oxygen through the decomposition of CuO_2_ under acidic conditions (Figure S15). Upon adding glucose, however, the generated oxygen was consumed by the enzymatic activity of GOx, enhancing the production of H_2_O_2_. Subsequently, we evaluated the H_2_O_2_ generation ability of HA‐PGMC under different conditions by incubating it in PBS at pH 7.4 or pH 5.5 containing 500 µm glucose (Figure 2B). Under acidic conditions, HA‐PGMC generated significantly higher levels of H_2_O_2_ compared to MC alone (Figure 1J), confirming the synergistic effect between CuO_2_ decomposition and GOx catalytic activity. Notably, HA‐PGMC also sustained robust H_2_O_2_ production under hypoxic (N_2_‐saturated) conditions (Figure S16), supporting its performance in the oxygen‐depleted tumor microenvironment where conventional O_2_‐dependent ROS therapies often fail. Next, we assessed the ability of HA‐PGMC to generate • OH using the TMB assay. 3,3′,5,5′‐Tetramethylbenzidine (TMB) serves as a substrate for colorimetric detection of •OHs, where •OH oxidizes TMB to form a blue‐colored product. The generation of •OH under different pH and glucose concentrations was evaluated (Figure 2C). It was found that HA‐PGMC produced •OHs only under dual conditions, pH 5.5 and 500 µm glucose, exhibiting a characteristic peak at ∼650 nm. Neither acidic pH nor glucose alone was sufficient to induce •OH production, suggesting a highly desirable tumor‐specific activation property to minimize damage to healthy tissues. We then investigated the time‐dependent generation of •OH by measuring absorbance at 10, 30 min, 1 h, and 4 h under pH 5.5 and 500 µm glucose (Figure 2D). The results demonstrated that •OH production was time dependent. Additionally, the concentration‐dependent generation of •OH was studied by incubating various concentrations of HA‐PGMC (0, 0.25, 0.50, 0.75, 1.00 mg/mL) in pH 5.5 and 500 µm glucose PBS (Figure 2E). Higher concentrations of HA‐PGMC led to greater •OH generation.

We next assessed the GSH depletion capability of HA‐PGMC using the Ellman's reagent (5,5′‐dithio‐bis‐(2‐nitrobenzoic acid), DTNB, which produces a yellow product upon reacting with GSH. The depletion was measured across pH (7.4 vs. 5.5) and glucose (0 vs. 500 µm) (Figure 2F). It was found that pH had a pronounced effect: greater depletion occurred at acidic pH, while glucose had minimal impact. This suggests that Cu^+^, generated from CuO_2_ decomposition under acidic conditions, oxidized GSH to GSSG. We further examined the time‐dependent GSH depletion ability of HA‐PGMC, finding that GSH depletion increased with longer incubation (0, 2, 4, 6 h) (Figure 2G). A concentration‐dependent trend was also evident, with higher HA‐PGMC levels producing stronger depletion (Figure 2H).

In summary, HA‐PGMC exhibits multiple favorable functions for cancer therapy. The H_2_O_2_ production from both CuO_2_ decomposition and GOx enzymatic activity was enhanced synergistically under acidic and glucose‐rich conditions. •OH generation is dual‐responsive to pH and glucose, and both time‐ and concentration‐dependent, occurring only under tumor‐relevant conditions. Meanwhile, GSH depletion is pH‐responsive and scales with time and concentration. Together, these properties indicate that HA‐PGMC can generate substantial ROS while concurrently disabling cellular antioxidant defences, providing a strong therapeutic advantage for amplifying oxidative stress within tumors.

### 2.3 Antitumor Efficacy of HA‐PGMC In Vitro

**FIGURE 3:**
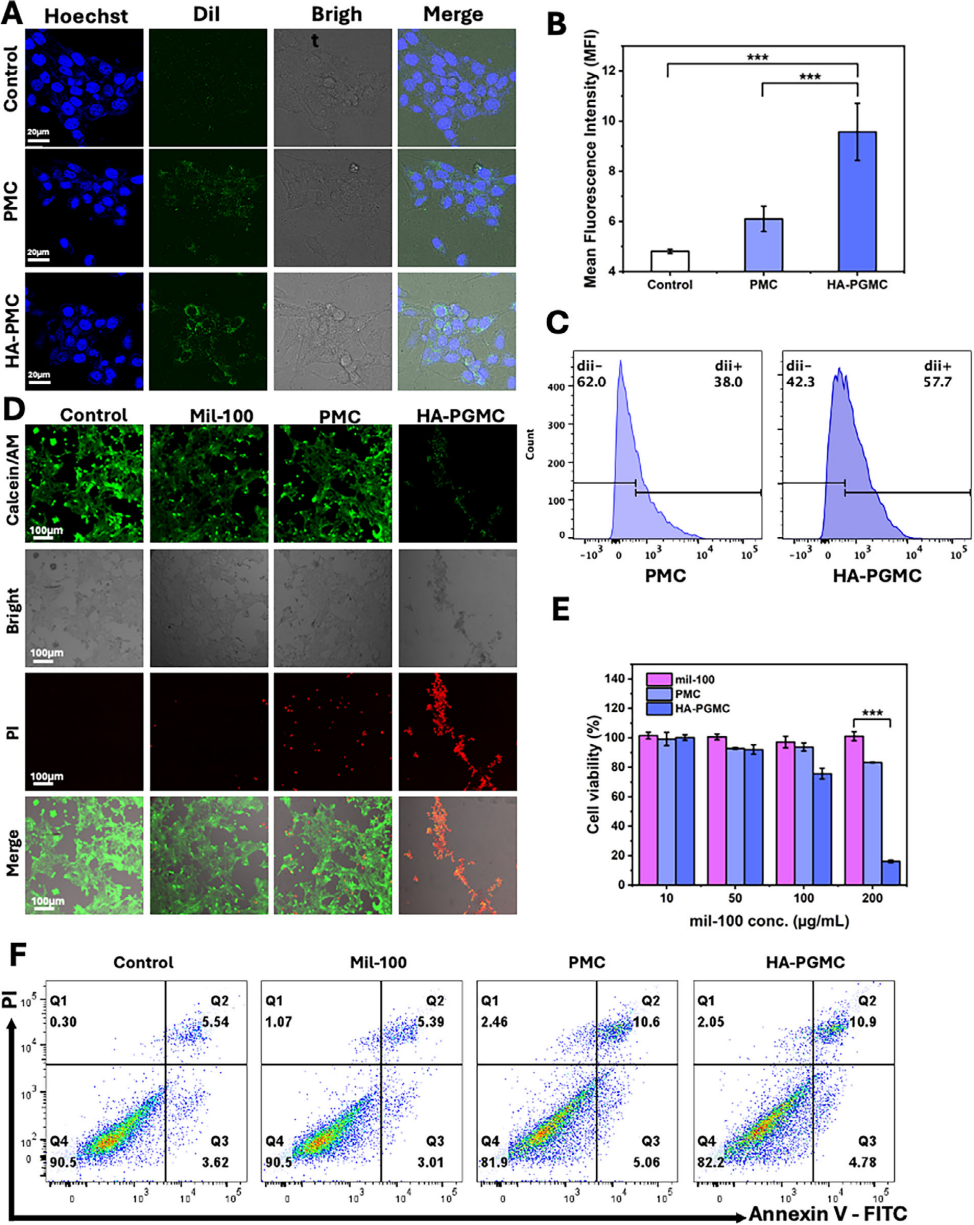
Antitumor efficacy of HA‐PGMC in vitro. (A) Confocal microscopy images of cellular uptake of DiI‐labelled PMC and HA‐PGMC in 4T1 cells. Blue: DAPI (nuclei); green: DiI‐labelled nanoparticles; grey: bright‐field. Scale bar = 20 µm. (B) Quantification of cellular uptake expressed as mean fluorescence intensity (MFI) of DiI in Control, PMC‐treated, and HA‐PGMC‐treated 4T1 cells. (C) Flow cytometry histograms showing the DiI^+^ population in PMC‐treated (left, 38.0%) and HA‐PGMC‐treated (right, 57.7%) 4T1 cells, confirming enhanced cellular uptake of the HA‐targeted nanoplatform. (D) Live/dead staining of 4T1 cells after 24 h incubation with different nanoparticles. Calcein‐AM (green) indicates live cells, propidium iodide (PI, red) indicates dead cells. Scale bar = 100 µm. (E) MTT cell viability assay of 4T1 cells treated with mil‐100, PMC, or HA‐PGMC at varying concentrations (10, 50, 100, and 200 µg/mL mil‐100 equivalent) for 24 h. (F) Annexin V/PI apoptosis assay of 4T1 cells following different treatments, showing progressive increases in early (Q3) and late (Q2) apoptotic populations from Control → mil‐100 → PMC → HA‐PGMC. Data are presented as mean ± SD (n = 3). Statistical significance: ^***^ *p* <0.001.

Encouraged by the favorable characterization of HA‐PGMC, we next evaluated its antitumor efficacy in vitro. First, cellular uptake was assessed in 4T1 cells by confocal laser scanning microscopy (CLSM) to verify targeting conferred by hyaluronic acid on PGMC. We pre‐labeled HA‐PGMC and PMC with the lipophilic dye DiI (Ex/Em 549/565 nm). After 4 h incubation with 4T1 cells, CLSM showed markedly stronger fluorescence for DiI‐HA‐PGMC than DiI‐PMC (Figure 3A). Quantitatively, HA‐PGMC exhibited approximately two‐fold higher fluorescence intensity than PMC, indicating HA‐mediated targeting (Figure 3B). To further quantify uptake at the population level, flow cytometry analysis of DiI‐labelled cells confirmed that HA‐PGMC achieved a higher DiI‐positive fraction (57.7%) compared to PMC (38.0%), providing direct evidence of enhanced CD44‐mediated cellular internalization (Figure 3C). We then evaluated antitumor activity in 4T1 cells using live/dead staining, MTT assay, and apoptosis analysis. Cell viability after 24 h nanoparticle exposure was assessed with Calcein AM/propidium iodide (PI) dual staining. CLSM revealed minimal Calcein AM and prominent PI fluorescence in PMC‐ and HA‐PGMC‐treated groups (Figure 3D), indicating strong cytotoxicity. In contrast, the mil‐100 group showed almost no red fluorescence, confirming that mil‐100 itself was non‐toxic to tumor cells. Consistent with these findings, MTT measurements showed concentration‐dependent viability loss for both HA‐PGMC and PMC, with HA‐PGMC reducing viability to ∼20% at 200 µg/mL, reflecting potent 4T1 killing (Figure 3E). Among all groups, mil‐100 maintained negligible cytotoxicity even at 200 µg/mL. We also examined cytotoxicity toward normal L929 fibroblasts (Figure S17). HA‐PGMC showed lower toxicity to normal cells than to tumor cells, maintaining ∼60% viability in L929, indicating preferential tumor selectivity.

Additionally, apoptosis was quantified to further validate the therapeutic effect. 4T1 cells were stained with Annexin V–FITC and PI after 24 h treatment and analyzed by flow cytometry. Results aligned with viability data: HA‐PGMC and PMC induced the highest apoptosis, reaching 10.9% and 10.6%, respectively (Figure 3F). In contrast, the mil‐100 group was comparable to the control, again supporting the high biocompatibility of mil‐100 as a carrier.

In summary, HA‐PGMC demonstrates excellent tumor‐targeting capability and potent antitumor efficacy, with high specificity toward tumor cells and minimal toxicity to normal cells, highlighting its promise for selective cancer therapy.

### 2.4 HA‐PGMC Functionalities In Vitro

We further assessed the functionalities HA‐PGMC in vitro, including GSH depletion, ROS generation, lipid peroxidation (LPO), and mitochondrial damage (JC‐1 assay), in 4T1 cells. The ability to deplete GSH and generate ROS is crucial for inducing ICD in tumors.

First, intracellular GSH was measured using an UV thiol tracker. As a tumor‐overexpressed antioxidant, GSH scavenges ROS and maintains redox homeostasis but limits CDT efficacy, which is an obstacle addressable by the Cu^2^^+^ ions released from HA‐PGMC. After 4 h incubation with the various nanoparticles, cells were stained with the thiol tracker and imaged by CLSM.

**FIGURE 4:**
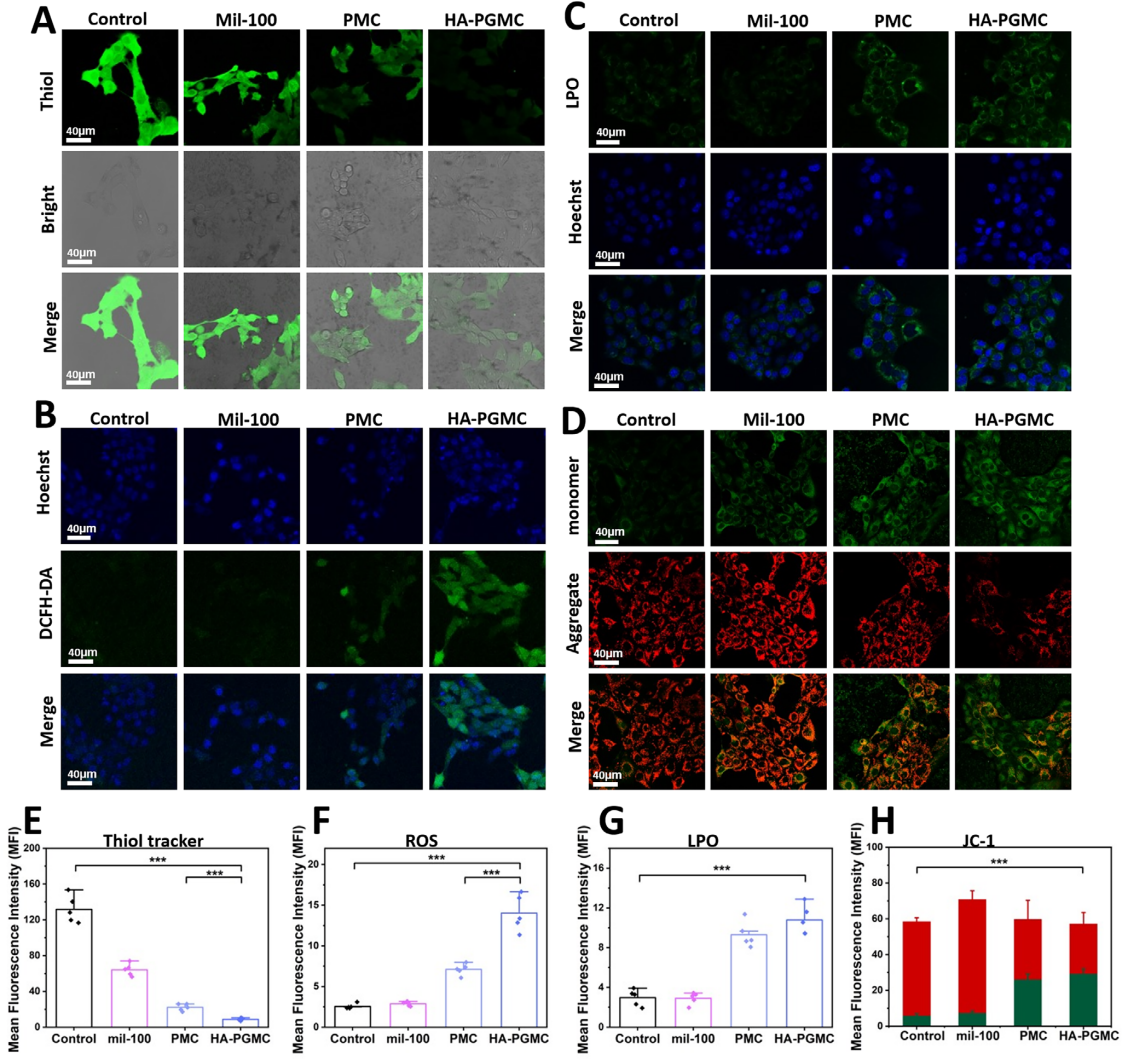
In vitro functionalities of HA‐PGMC were assessed in 4T1 cells, including (A) GSH depletion using thiol tracker, (B) ROS generation with DCFH‐DA, (C) lipid peroxidation via LPO probe, and (D) mitochondrial membrane potential disruption through JC‐1 assay. Quantified fluorescence intensities are shown in (E–H). Scale bar = 40 µm. Data are presented as mean ± SD (n = 5). Statistical significance was determined by one‐way ANOVA with Tukey's post‐hoc test (^*^ *p* <0.05, ^**^ *p* <0.01, ^***^ *p* <0.001).

As shown in Figure 4A, both control and mil‐100 groups displayed strong green fluorescence (thiol tracker), whereas HA‐PGMC and PMC showed negligible signal. Notably, the mean fluorescence of mil‐100 was significantly lower than that of the control, while HA‐PGMC was the lowest among all (Figure 4E). An additional thiol tracker image with Hoechst nuclear counterstain further confirmed the intracellular localization of the depleted thiol signal and the corresponding nuclear morphology across treatment groups (Figure S18). This could be attributed to the fact that Fe^2^^+^ in mil‐100 also participates in GSH depletion via Fenton reactions, whereas HA‐PGMC generated more H_2_O_2_ to facilitate Cu^+^‐mediated oxidation of GSH. Next, we evaluated the ROS generation ability of the nanoparticles using 2',7'‐dichlorodihydrofluorescein diacetate (DCFH‐DA). After 4 h treatment, the HA‐PGMC group exhibited the strongest fluorescence signal, followed by PMC (Figure 4B), while mil‐100 and control showed minimal ROS. Quantitatively, HA‐PGMC induced nearly double the fluorescence of PMC (Figure 4F), confirming that GOx‐derived H_2_O_2_ markedly amplifies ROS generation. This trend was further corroborated by DCFH‐DA flow cytometry analysis, in which HA‐PGMC‐treated cells displayed the highest population‐level DCF fluorescence compared with PMC and control, providing quantitative confirmation of enhanced intracellular ROS generation by HA‐PGMC (Figure S19). We then assessed the level of lipid peroxidation (LPO), a key indicator of ROS damage to membranes. After 4 h incubation and staining with an LPO probe, HA‐PGMC yielded the strongest green fluorescence, indicating the highest LPO (Figure 4C), with PMC showing comparable intensity and mil‐100/control remaining low (Figure 4G).

Finally, mitochondrial membrane potential (MMP) disruption was examined using JC‐1. Healthy mitochondria exhibit red J‐aggregates, while depolarized mitochondria display green monomers. HA‐PGMC markedly increased green fluorescence signal, indicating severe mitochondrial dysfunction driven by ROS (Figure 4D). PMC caused a moderate increase in green fluorescence signal, whereas mil‐100 and control remained predominantly red (Figure 4H).

Collectively, these results confirm that HA‐PGMC efficiently depletes intracellular GSH, overcoming one of the major limitations of CDT, and dramatically enhances oxidative stress through synergistic ROS production from GOx, pH‐responsive H_2_O_2_ release from CuO_2_, and Fe–Cu catalytic cascade reactions. The heightened oxidative stress drives extensive lipid peroxidation and mitochondrial dysfunction, thereby promoting tumor cell death.

### 2.5 HA‐PGMC Induced ICD In Vitro

ROS‐induced immunogenic cell death (ICD) stimulates the release of damage‐associated molecular patterns (DAMPs), including calreticulin (CRT), high‐mobility group box 1 (HMGB1), and adenosine triphosphate (ATP), from dying tumor cells. These DAMPs promote the maturation of dendritic cells (DCs), recruit T lymphocytes, and initiate an antitumor immune response, effectively converting “cold” tumors to “hot,” immunogenic states.

**FIGURE 5:**
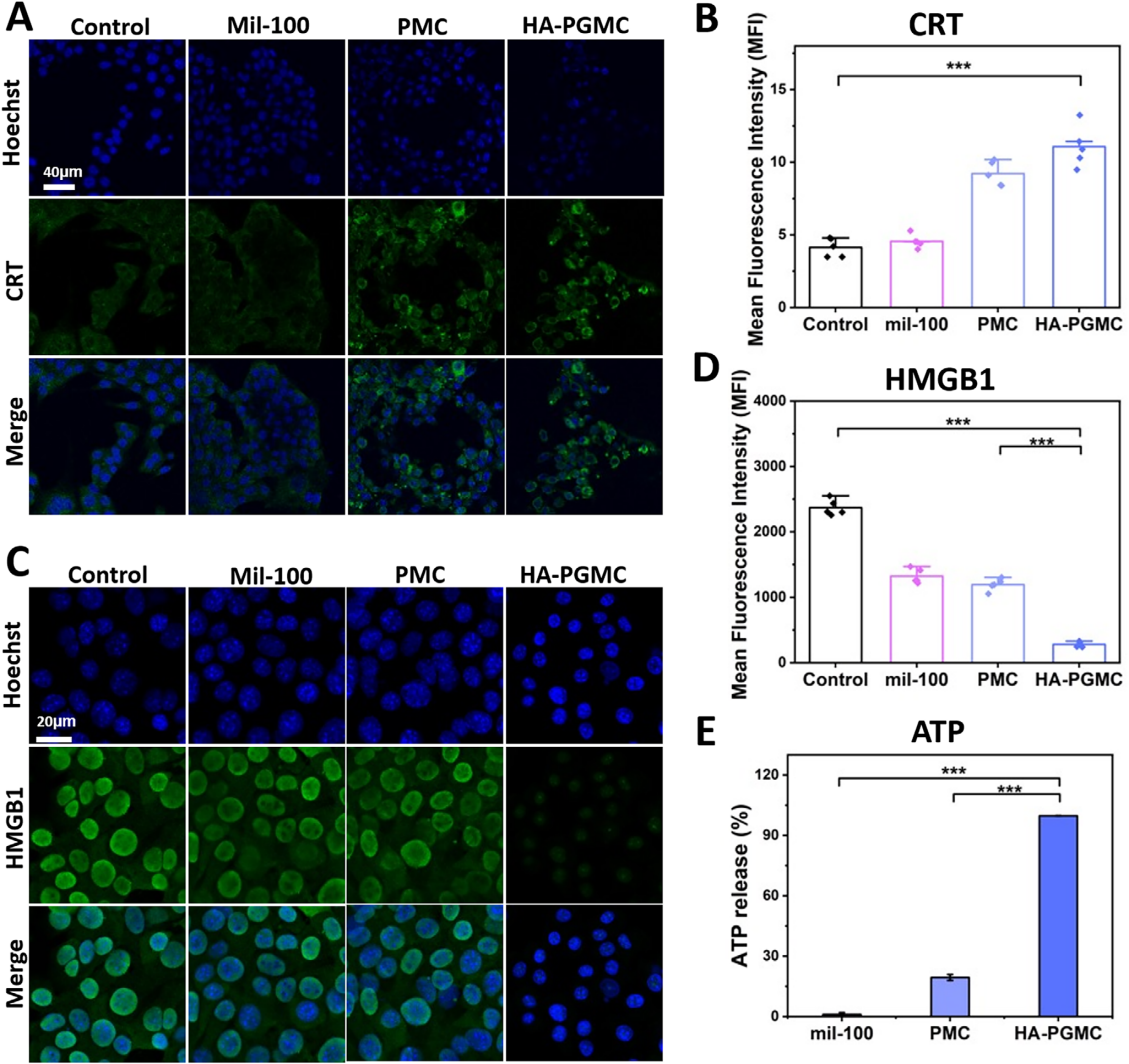
HA‐PGMC‐induced immunogenic cell death (ICD) in vitro. (A) CRT and (C) HMGB1 after treatment with different nanoparticles. Scale bars: 40 µm (A) and 20 µm (C). Quantitative analysis of fluorescence intensities is shown in (B,D), respectively. (E) ATP release from 4T1 cells after treatment with different nanoparticles. Data are presented as mean ± SD (n = 5). Statistical significance was determined by one‐way ANOVA with Tukey's post‐hoc test (^*^ *p* <0.05, ^**^ *p* <0.01, ^***^ *p* <0.001).

To assess the ability of HA‐PGMC to induce ICD, 4T1 cells were incubated with different nanoparticles for 24 h and then stained with CRT and HMGB1 antibodies conjugated with fluorophores, allowing visualization by confocal laser scanning microscopy. First, we evaluated CRT exposure on the surface of 4T1 cells. As shown in Figure 5A, cells treated with HA‐PGMC and PMC displayed intense green fluorescence, while the mil‐100 treated group exhibited negligible signal. Quantitative analysis confirmed that the mean fluorescence intensity of HA‐PGMC was the highest among all groups (Figure 5B), suggesting a stronger ability to trigger CRT exposure. Next, we investigated the release of HMGB1, a hallmark of late‐stage ICD. Unlike CRT, which accumulates on the cell surface upon ICD induction, HMGB1 is normally retained within the nucleus under basal conditions and is actively secreted into the extracellular space during ICD — resulting in a decrease in intracellular fluorescence signal. As shown in Figure 5C, untreated control cells exhibited strong intracellular HMGB1 green fluorescence, consistent with its nuclear localization under non‐stimulated conditions. In contrast, cells treated with HA‐PGMC showed markedly reduced intracellular HMGB1 fluorescence, indicating substantial extracellular translocation of HMGB1 as a consequence of ICD. The mil‐100 and PMC groups exhibited intermediate levels of intracellular HMGB1 fluorescence, suggesting comparatively weaker ICD induction. Quantification further confirmed that the HA‐PGMC group exhibited the lowest mean fluorescence intensity among all groups (Figure 5D), consistent with the greatest extent of HMGB1 extracellular release, followed by the PMC group. Consistent with the CRT and HMGB1 results, HA‐PGMC treatment led to a significant increase in ATP release from 4T1 cells, reaching 99%—approximately five times higher than that observed in the PMC‐treated group (Figure 5E). In contrast, the mil‐100 group showed minimal ATP release.

To orthogonally validate these findings at the whole‐protein level, Western blot analysis was performed on 4T1 cells treated with Control, mil‐100, PMC, and HA‐PGMC. Consistent with the confocal imaging results, HA‐PGMC‐ and PMC‐treated cells showed markedly elevated CRT exposure and reduced intracellular HMGB1 (indicating nuclear‐to‐cytosolic translocation and subsequent extracellular release), while mil‐100 and Control displayed baseline levels (Figure S20). This dual‐modality confirmation further supports HA‐PGMC‐induced immunogenic cell death.

Together, these findings demonstrate that HA‐PGMC robustly induces ICD by promoting CRT exposure, HMGB1 release, and ATP secretion, underscoring its strong potential to convert immunosuppressive “cold tumors” into inflamed “hot tumors” and thereby enhance the efficacy of immunotherapy.

### 2.6 In Vivo Biodistribution

Building on the promising in vitro antitumor efficacy, we assessed in vivo performance of HA‐PGMC in a 4T1 murine breast cancer xenograft model, combined with the immune checkpoint inhibitor αPD‐L1. To evaluate targeting, nanoparticles were labeled with the near‐infrared dye DiR (1,1′‐dioctadecyl‐3,3,3′,3′‐tetramethylindotricarbocyanine iodide; Ex/Em 750/780 nm). 4T1 tumor‐bearing mice were assigned to two groups: (1) DiR–PMC and (2) DiR–HA‐PGMC. Whole‐body fluorescence was monitored by IVIS at 0, 4, 8, 12, and 24 h, followed by ex vivo organ imaging.

**FIGURE 6:**
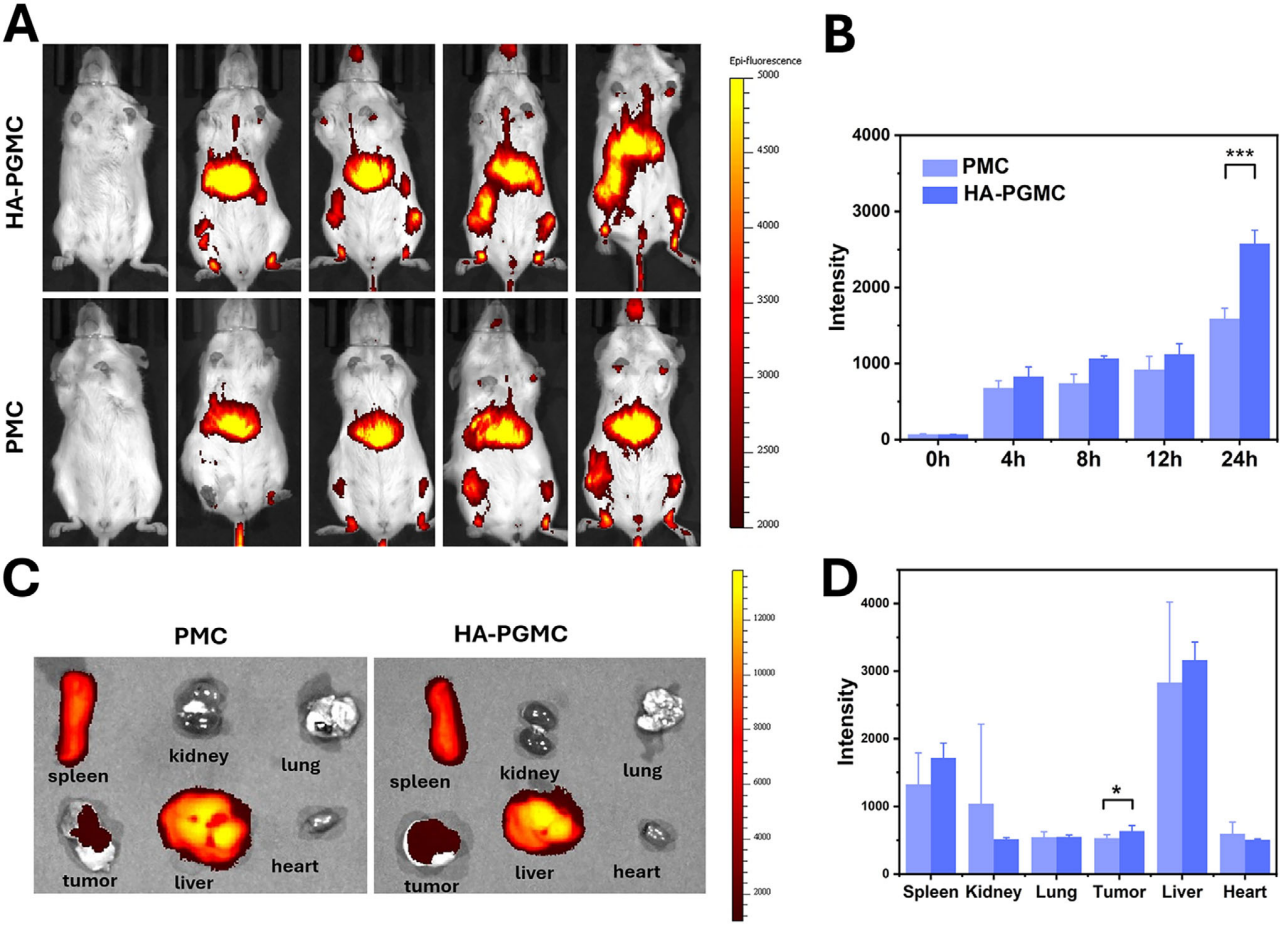
In vivo biodistribution. (A) fluorescence image of mice of different treatments (PMC and HA‐PGMC) using IVIS, and (B) corresponding fluorescence intensity of different treatment groups. (C) ex vivo image of organs and tumors after different treatments, and (D) corresponding fluorescence intensity of different treatment groups in organs and tumors. Data are presented as mean ± SD (n = 4). Statistical significance was determined by one‐way ANOVA with Tukey's post‐hoc test (^*^ *p* <0.05, ^**^ *p* <0.01, ^***^ *p* <0.001).

After intravenous injection of DiR‐labeled nanoparticles, the HA‐PGMC group consistently exhibited stronger tumor fluorescence compared with the PMC group (Figure 6A). Notably, at 24 h post‐injection, the fluorescence intensity of HA‐PGMC in tumors was approximately 60% higher than that of PMC (Figure 6B). Ex vivo imaging of excised tumors and major organs corroborated these findings (Figure 6C), and quantitative analysis further confirmed the enhanced tumor accumulation of HA‐PGMC (Figure 6D). Importantly, HA‐PGMC displayed markedly lower fluorescence in the liver and spleen compared with PMC, suggesting reduced reticuloendothelial uptake and improved tumor‐specific accumulation.

Collectively, these results highlight the superior tumor‐targeting capacity of HA‐conjugated nanoparticles, a key attribute for boosting therapeutic efficacy while minimizing systemic side effects.

### 2.7 In Vivo Antitumor Efficacy

**FIGURE 7:**
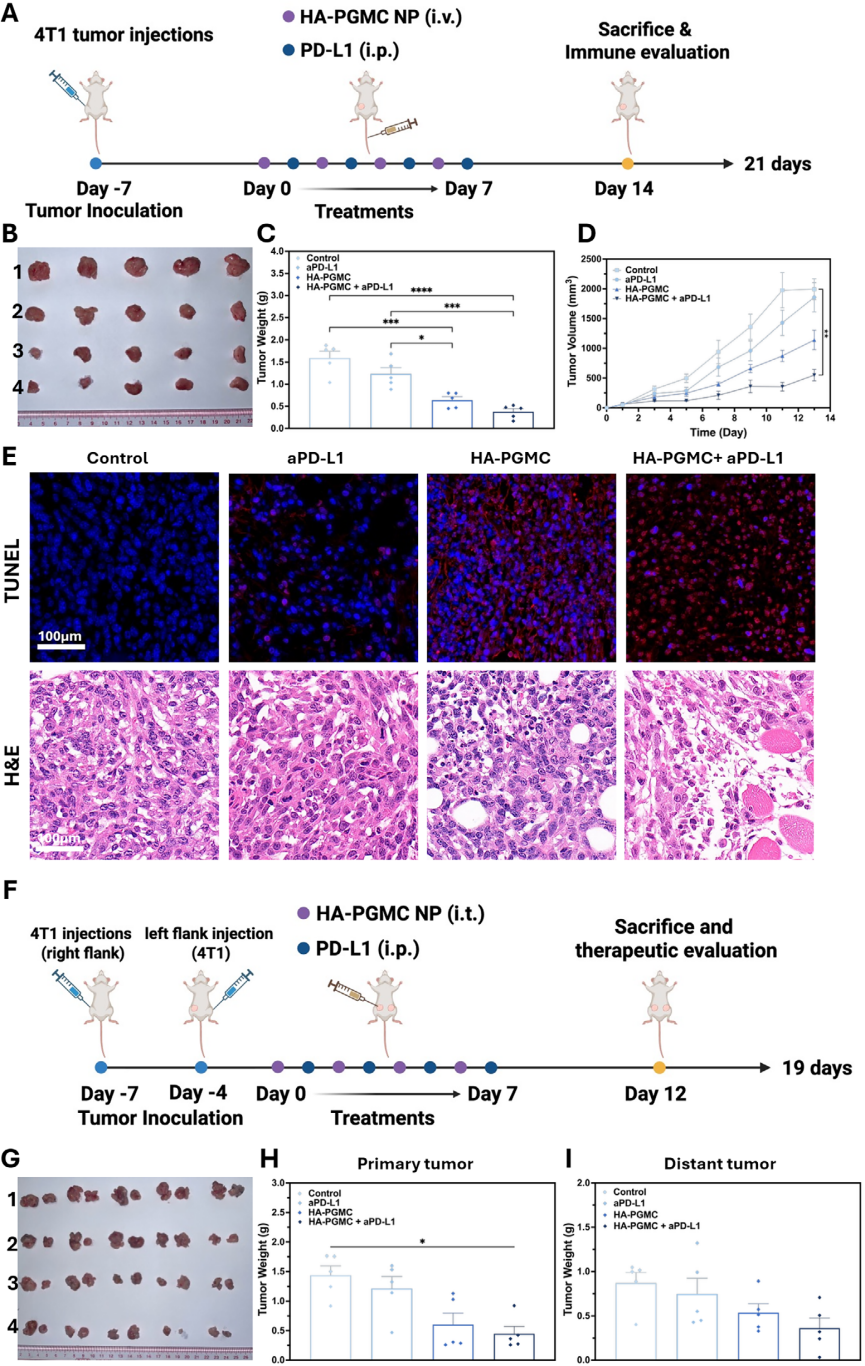
In vivo antitumor efficacy of HA‐PGMC. (A) Single‐tumor treatment schedule: 4T1 inoculation on Day −7; i.v. HA‐PGMC and i.p. αPD‐L1 every 2 days × 4 cycles (Days 0–7); sacrifice on Day 14. Groups: (1) Control, (2) αPD‐L1, (3) HA‐PGMC, (4) HA‐PGMC + αPD‐L1. (B) Representative images of excised tumors. (C) Tumor weights and (D) tumor volume growth curves. (E) Representative TUNEL (upper; red: apoptotic nuclei; blue: DAPI) and H&E (lower) staining of tumor sections. Scale bar = 100 µm. (F) Bilateral tumor model: primary tumor (right, Day −7), distant tumor (left, Day −4); i.t. HA‐PGMC + i.p. αPD‐L1 (Days 0–7); sacrifice on Day 12. (G) Representative images of excised primary and distant tumors. (H) Primary tumor weights and (I) distant tumor weights. Data are presented as mean ± SD (n = 5). Statistical analysis was performed using one‐way ANOVA followed by Tukey's post‐hoc test for normally distributed data (verified by the Shapiro–Wilk test), or Kruskal–Wallis test followed by Dunn's test for non‐normally distributed data. (^*^ *p* <0.05, ^**^ *p* <0.01, ^***^ *p* < 0.001).

After confirming the tumor targeting capability of HA‐PGMC, we next evaluated its therapeutic efficacy in vivo using a 4T1 breast tumor xenograft model. BALB/c mice bearing tumors of ∼60–80 mm^3^ were randomly divided into four groups: (1) PBS, (2) αPD‐L1, (3) HA‐PGMC, and (4) HA‐PGMC + αPD‐L1. Following subcutaneous tumor inoculation and a 7‐day growth period, mice received the first intravenous dose of nanoparticles or PBS (Figure 7A), followed by αPD‐L1 intraperitoneal injection the next day. Treatments were administered every 2 days for four cycles (Days 0, 2, 4, and 6 for nanoparticles; Days 1, 3, 5, and 7 for αPD‐L1). Mice were sacrificed on Day 14 post‐initial‐treatment (Day 21 post‐inoculation) to assess therapeutic efficacy and immune activation.

As shown in Figure 7B, the combination of HA‐PGMC + αPD‐L1 yielded the most pronounced inhibition of tumor growth compared with all other groups, highlighting a clear synergistic effect. This was further supported by reductions in tumor burden: both tumor weight and tumor volume were substantially reduced in the combination group relative to controls (Figure 7C,D). Individual tumor volume trajectories for each animal (Figure S23) and group‐averaged mean tumor volume curves over the 14‐day treatment window (Figure S24) further confirmed the consistent and sustained tumor suppression achieved by HA‐PGMC + αPD‐L1 across all treated mice. Specifically, the average tumor weight in the HA‐PGMC + αPD‐L1 group decreased by nearly 70% versus PBS, while tumor volume was suppressed to ∼22% of control size. Importantly, the monotherapy groups (HA‐PGMC or αPD‐L1 alone) also showed measurable tumor inhibition, but their efficacy was consistently lower than that observed with the combination treatment.

To further validate the therapeutic efficacy, histology was performed using hematoxylin and eosin (H&E) staining and TUNEL assays to assess tissue damage and apoptosis. H&E revealed extensive necrosis in the HA‐PGMC + αPD‐L1 group compared with others (Figure 7E). Consistently, TUNEL assays demonstrated markedly higher levels of apoptosis in the combination group (Figure 7E). Notably, HA‐PGMC treatment alone induced greater apoptotic cell death than αPD‐L1 monotherapy, underscoring the superior therapeutic potential of the nanoplatform and its synergistic benefit with checkpoint blockade.

To further evaluate whether HA‐PGMC‐induced immune activation could elicit a systemic antitumor response, we established a bilateral tumor model. A primary tumor was inoculated on the right flank (Day −7), followed by a distant tumor on the left flank (Day −4). HA‐PGMC was administered via intratumoral (i.t.) injection into the primary tumor only, with αPD‐L1 given intraperitoneally from Day 0 to Day 7. Mice were sacrificed on Day 12 post‐treatment (Day 19 post‐inoculation) (Figure 7F). As shown in Figure 7G,H, the combination of HA‐PGMC + αPD‐L1 significantly suppressed primary tumor growth compared with all other groups, consistent with the single‐tumor model results. Critically, the distant untreated tumors also exhibited marked growth inhibition in the combination group (Figure 7I), demonstrating a robust abscopal effect. Complementary quantitative analyses — including combined tumor weight, individual primary and distant tumor growth curves, and total tumor burden — are summarized in Figure S25A–D, which collectively reinforce the synergistic dual‐tumor suppression of HA‐PGMC + αPD‐L1. This finding provides direct evidence that HA‐PGMC‐induced immunogenic cell death, in combination with immune checkpoint blockade, can trigger systemic antitumor immunity capable of controlling distant, untreated tumors.

Taken together, these results demonstrate that HA‐PGMC, when combined with immune checkpoint blockade, exerts potent synergistic antitumor effects in both local and distant tumors, effectively suppressing tumor progression in vivo. The observed abscopal effect further underscores the potential of combining CDT‐based nanoplatforms with immunotherapy to overcome tumor immune evasion and achieve durable therapeutic outcomes.

### 2.8 In Vivo Immune Evaluation

After 14 days of treatment, tumors were harvested, enzymatically digested into single‐cell suspensions, and analyzed by flow cytometry to assess immune activation. In 4T1 tumor‐bearing mice, ROS‐driven immunogenic cell death induced by HA‐PGMC, especially when combined with αPD‐L1, produced pronounced immunomodulatory effects.

**FIGURE 8:**
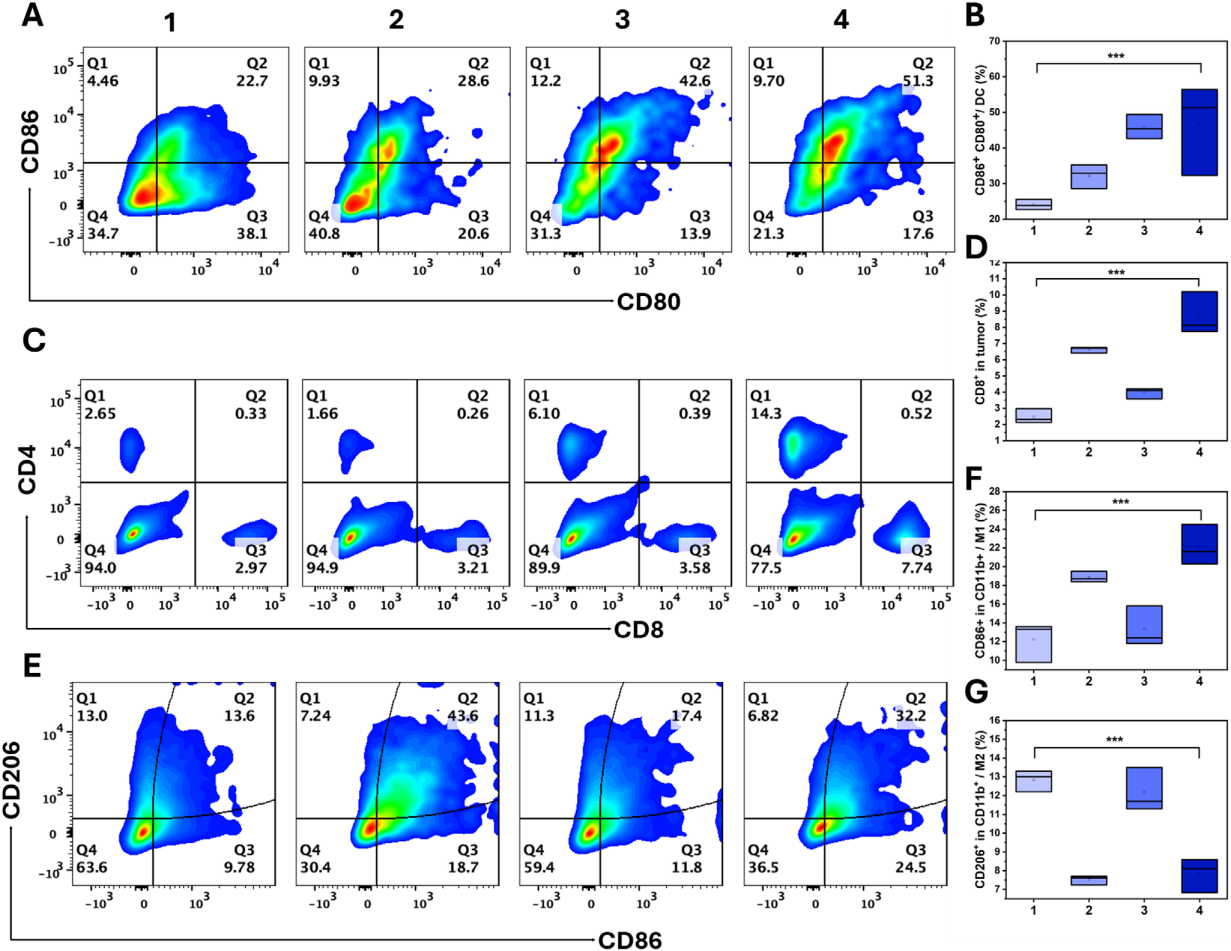
In vivo immune evaluation. (A) flow cytometric assay of DCs in tumor and (B) the corresponding quantification of DCs (1: Control, 2: αPD‐L1, 3: HA‐PGMC, 4: HA‐PGMC + αPD‐L1). (C) flow cytometric assay of CD8+ cells in tumor and (D) the corresponding quantification of CD8+ cells. (E) flow cytometric assay of M1 and M2 macrophages in tumor and (F) the corresponding quantification of M1 macrophages, (G) the corresponding quantification of M2 macrophages. Data are presented as mean ± SD (n = 5). Statistical significance was determined by one‐way ANOVA with Tukey's post‐hoc test (^*^ *p* <0.05, ^**^ *p* <0.01, ^***^ *p* <0.001).

Specifically, HA‐PGMC treatment increased the proportion of mature dendritic cells (DCs) from 22.7% in the control group to 42.6%. The combination of HA‐PGMC with αPD‐L1further enhanced DC maturation to 51.3% (Figure 8A), underscoring its strong potential to prime antitumor T‐cell responses (Figure 8B).

T‐cell infiltration was also markedly improved. The HA‐PGMC + αPD‐L1group showed the highest proportion of CD8^+^ tumor‐infiltrating lymphocytes (TILs) at 7.74%, approximately 2.5‐fold higher than the control (Figure 8C,D). In parallel, CD4^+^ helper T cells were significantly elevated in the HA‐PGMC group, reaching 14.3%, a nearly fivefold increase compared with controls (Figure S26). To further characterize the immunosuppressive compartment within the tumor, tumor‐infiltrating regulatory T cells (Tregs) were profiled by flow cytometry using CD45^+^CD4^+^FoxP3^+^ gating. Representative dot plots are shown in Figure S27. Quantitative analysis revealed no significant differences in Treg frequency across treatment groups (Figure S28), indicating that the antitumor efficacy of HA‐PGMC + αPD‐L1 is primarily driven by enhanced effector T‐cell recruitment and activation (CD8^+^ TIL expansion and DC maturation) rather than by direct depletion of the Treg population.

Macrophage polarization analysis further revealed a shift toward a pro‐inflammatory phenotype (Figure 8E). HA‐PGMC treatment reprogrammed tumor‐associated macrophages into the M1 phenotype, reaching 24.5% (2.5‐fold above control; Figure 8F), while simultaneously suppressing the immunosuppressive M2 subtype to 6.82%, nearly half the control level (Figure 8G).

Collectively, these findings demonstrate that HA‐PGMC, particularly in combination with αPD‐L1, orchestrates a multifaceted immune response by enhancing DC maturation, promoting CD8^+^ and CD4^+^ T‐cell infiltration, and reprogramming macrophages from M2 to M1. This synergy not only initiates robust antitumor immunity but also remodels the immunosuppressive tumor microenvironment into a pro‐inflammatory, immune‐active state.

### 2.9 In Vivo Biosafety

The biosafety of the different treatments was further evaluated through blood biochemical analysis and histological examination. Key serum markers, including alanine aminotransferase (ALT), aspartate aminotransferase (AST), creatine kinase (CK), blood urea nitrogen (UREA), and creatinine (CREA), were measured after 14 days of treatment. No significant differences were observed among the treatment groups, indicating normal liver, kidney, and cardiac function (Figure S29). In parallel, histological analysis of major organs using H&E staining revealed no detectable abnormalities or tissue damage across all groups (Figure S30). To further characterize the organ distribution of injected nanoparticles, Cu and Fe content in major organs and tumor tissue was quantified by ICP‐OES at 24 h post‐injection. Both PMC and HA‐PGMC groups showed comparable metal accumulation profiles, with primary deposition in liver and spleen consistent with standard nanoparticle clearance via the reticuloendothelial system, and no excessive off‐target accumulation in heart, kidney, or lung (Figures S21 and S22). Collectively, these findings demonstrate that the HA‐PGMC–based treatment exhibits excellent biosafety, supporting its potential as a safe and effective therapeutic strategy with negligible systemic side effects.

## 3 Conclusion

In summary, we have developed a multifunctional ROS self‐supplying nanoplatform (HA‐PGMC) that integrates mil‐100, in situ‐grown CuO_2_ nanodots, GOx, PEG‐PLA, and HA to achieve efficient chemodynamic and immunotherapy. By leveraging dual H_2_O_2_ generation and a Cu─Fe cascade catalytic cycle, HA‐PGMC effectively amplifies ROS production, overcomes GSH‐mediated resistance, and induces robust immunogenic cell death. Both in vitro and in vivo studies demonstrated enhanced tumor accumulation, potent antitumor efficacy, and multifaceted immune activation, particularly when combined with αPD‐L1 immune checkpoint blockade. In a bilateral 4T1 tumor model, the combination therapy not only suppressed the treated primary tumor but also significantly inhibited the growth of distant, untreated tumors, demonstrating a robust abscopal effect and confirming that HA‐PGMC‐induced ICD can trigger systemic antitumor immunity. Importantly, HA‐PGMC successfully reprogrammed immunologically “cold” tumors into “hot” tumors, thereby addressing a critical limitation of current ICB therapies.

These findings underscore the promise of HA‐PGMC as a next‐generation nanoplatform that not only provides tumor‐specific, hypoxia‐tolerant ROS amplification but also potentiates both local and systemic antitumor immunity. With its reproducible one‐pot synthesis, favorable biocompatibility profile, and demonstrated synergy with immune checkpoint blockade, this strategy offers considerable potential for clinical translation in the treatment of aggressive cancers such as TNBC.

## 4 Experimental Section

### 4.1 Materials and Reagents

Iron(III) chloride hexahydrate was obtained from Sigma–Aldrich. Copper chloride dihydrate and trimesic acid were obtained from Macklin. Polyvinylpyrrolidone (PVP, MW 10 000) was obtained from Sigma–Aldrich. Poly(ethylene glycol)‐OH (mPEG‐OH, MW 2000) was purchased from Aladdin. D, L‐lactide was acquired from TCI Chemicals. Glucose oxidase (GOx) and hyaluronic acid (HA) were obtained from Macklin. H_2_O_2_ assay kit was obtained from Beyotime. Cell culture media, including Dulbecco's Modified Eagle Medium (DMEM), fetal bovine serum (FBS), and penicillin‐streptomycin, were purchased from Thermo Fisher Scientific. For fluorescence imaging and nuclear staining, Hoechst 33342 and DiI dyes were obtained from Beyotime. Viability assays were conducted using the Live/Dead staining kit, MTT reagent, and apoptosis detection kit, all from Beyotime Biotechnology. Reactive oxygen species (ROS) levels were evaluated using DCFH‐DA (Beyotime), while intracellular glutathione (GSH) was detected using Thiol Tracker Violet (AAT Bioquest). Lipid peroxidation was measured with a kit from Dojindo Laboratories. The mitochondrial membrane potential was assessed via the JC‐1 assay kit (Beyotime). For immunogenic cell death (ICD) evaluation, antibodies targeting HMGB1 and CRT were sourced from BioLegend and Servicebio, respectively. Finally, extracellular ATP release was quantified using an assay kit from Beyotime.

### 4.2 Characterization

Transmission electron microscopy (TEM) images were acquired using a FEI TS12 instrument. The hydrodynamic diameter and surface charge (zeta potential) of the particles were determined with a Malvern Zetasizer ZS90. Optical absorbance in biochemical assays was measured using a Multiskan FC plate reader (Thermo Scientific). The level of dissolved oxygen was evaluated with an AR8406 DO meter (IntellSMART). Surface chemical composition was analyzed via X‐ray photoelectron spectroscopy (XPS) on a Thermo Scientific Escalab 250Xi system. Confocal fluorescence imaging was conducted using a Leica TCS SP8 confocal microscope. Flow cytometry measurements were performed on a BD Symphony A5.2 SORP platform. Copper ion concentration was quantified using inductively coupled plasma optical emission spectrometry (ICP‐OES, Shimadzu ICPE‐9820). The crystalline structure of the samples was characterized through X‐ray diffraction (XRD) using a Rigaku SmartLab 9 kW diffractometer (HD2931N model).

### 4.3 Synthesis of mil‐100

1.68 g Iron(III) chloride hexahydrate was mixed with 0.54 g trimesic acid in 20 mL DI water. Acetic acid (0.16 mL) was added to the mixture and stirred for 30 min. The mixture was digested under microwave digestion at 120 °C for 5 min. The resulting solution was centrifuged at 6000 rpm for 5 min, and the supernatant was reserved. The nanoparticles were washed three times with DI water and stored in the fridge.

### 4.4 Synthesis of MC

16 mg mil‐100 was mixed with 34 mg Copper chloride dihydrate and 0.2 g PVP in 20 mL of water. 20 mL 0.02 m NaOH solution was added into the mixture, followed by adding 0.4 mL H_2_O_2_ (30%) dropwise. The reaction was maintained for 30 min, and then MC was washed with EtOH three times.

### 4.5 Synthesis of PEG_44_‐PLA_95_ Copolymer

To eliminate residual moisture, methoxy poly(ethylene glycol) (mPEG‐OH, 0.2 mg, MW 2000) and D, L‐lactide (1.3 g) were co‐evaporated in 50 mL of anhydrous toluene. Following this dehydration step, tin(II) 2‐ethylhexanoate (Sn(Oct)_2_, 0.1 mL) was introduced as the catalyst, and the reaction mixture was purged with nitrogen gas for 30 min to ensure an inert atmosphere. The ring‐opening polymerization was then conducted at 120 °C for 24 h in 20 mL of toluene under nitrogen protection. After the reaction was complete, the product was isolated by precipitation in chilled diethyl ether and subsequently characterized by nuclear magnetic resonance (NMR) spectroscopy. 1H NMR (500 MHz, Chloroform‐d) δ 5.25 – 5.11 (m, 212H), 3.65 (s, 176H), 3.38 (s, 3H), 1.62 – 1.51 (m, 636H).

### 4.6 Synthesis of PMC

16 mg MC and 30 mg PEG‐PLG were mixed in 4 mL THF, followed by dropping into 20 mL DI water under sonication. The mixture was evaporated under 50 °C for 5 min and washed with DI water for three times.

### 4.7 Synthesis of HA‐PGMC

8 mg PMC was mixed with 8 mg HA, 0.8 g GOx in 5 mL DI water. The mixture was stirred for 1.5 h. The resulting HA‐PGMC was washed three times using DI water.

### 4.8 Cu & Fe Release

200 µL HA‐PGMC (1 mg/mL) were distributed into different conditions: (1) 0 µm glucose, 7.4 PBS, (2) 500 µm glucose, 7.4 PBS, (3) 0 µm glucose, 5.5 PBS, (4) 500 µm glucose, 5.5 PBS. At predetermined timepoints (1, 3, 7, 10, and 24 h), HA‐PGMC was centrifuged, and 100 µL supernatant was extracted. 100 µL corresponding buffer was added back to the tubes. The Cu & Fe release were measured using ICP‐OES.

### 4.9 H_2_O_2_ Quantification

200 µL 100ug/mL HA‐PGMC and MC were placed into different conditions to test the release of H_2_O_2_, where MC was placed into PBS with different pH (7.4 & 5.5), and HA‐PGMC was placed into 500 µm glucose PBS with different pH. After an hour of incubation in 37 °C, 100 µL supernatant was extracted and measured the H_2_O_2_ release. The measurement was conducted under manufacturer's instructions.

### 4.10 Dissolved Oxygen of HA‐PGMC and MC

5 mg HA‐PGMC and MC were placed into 5 mL of different conditions for confirming the oxygen level after reaction, where MC was placed into PBS in 5.5 & 7.4, and HA‐PGMC was placed into PBS in 5.5 with 500 µm glucose. The dissolved oxygen was measured at predetermined timepoints (5, 10, 15, 20, 25, and 30 min).

### 4.11 TMB Assay on •OH Generation

200 µL HA‐PGMC was distributed into PBS buffer with TMB (5 mg/mL). Three different scenarios were considered: (1) condition‐dependent, (2) time‐dependent, and (3) concentration‐dependent. For (1), HA‐PGMC was incubated with TMB in different PBS, including (a) 0 µm glucose, 7.4 PBS, (b) 500 µm glucose, 7.4 PBS, (c) 0 µm glucose, 5.5 PBS, (d) 500 µm glucose, 5.5 PBS. For (2), HA‐PGMC was incubated with TMB in 500 µm glucose, 5.5 PBS, and •OHs were measured at predetermined timepoints. For (3), different concentrations of HA‐PGMC were incubated with TMB in 500 µm glucose, 5.5 PBS.

### 4.12 DTNB Assay on GSH Depletion

200 µL HA‐PGMC was distributed into PBS buffer with GSH (10 mL). Three different scenarios were considered: (1) condition‐dependent, (2) time‐dependent, and (3) concentration‐dependent. For (1), HA‐PGMC was incubated with GSH in different PBS, including (a) 0 µm glucose, 7.4 PBS, (b) 500 µm glucose, 7.4 PBS, (c) 0 µm glucose, 5.5 PBS, (d) 500 µm glucose, 5.5 PBS. For (2), HA‐PGMC was incubated with GSH in 500 µm glucose, 5.5 PBS, and GSH depletion were measured at predetermined timepoints. For (3), different concentrations of HA‐PGMC were incubated with GSH in 500 µm glucose, 5.5 PBS.

### 4.13 Cell Culture

The L929 mouse fibroblasts and 4T1 murine breast cancer cells were grown in Dulbecco's Modified Eagle Medium (DMEM) containing 10% fetal bovine serum and 1% penicillin–streptomycin. Cultures were incubated at 37 °C in a humidified environment with 5% CO_2_.

### 4.14 Cellular Uptake

To evaluate cellular uptake, 4T1 cells were plated in eight‐well chamber slides at a density of 2.0 × 10^4^ cells per well and allowed to adhere overnight. The following day, cells were treated with DiI‐labeled nanoparticles, including PMC and HA‐PGMC (20 µg/mL) diluted in complete medium and incubated for 4 h. After exposure, the cells were rinsed three times with PBS and stained with Hoechst 33342 (1 µg/mL) for 10 min. Fluorescence images were then acquired using a confocal laser scanning microscope.

### 4.15 Live/Dead Staining

4T1 cells were seeded in eight‐well confocal chamber slides at a density of 0.2 × 10^5^ cells per well and incubated overnight to allow cell adhesion. The next day, cells were exposed to different nanoparticle formulations (200 µg/mL) for 24 h. Post‐treatment, live/dead cell staining was performed using Calcein‐AM and propidium iodide (PI) for 30 min. The samples were then imaged with a confocal microscope to evaluate cell viability based on fluorescence signals.

### 4.16 Cell Cytotoxicity Assay

4T1 and L929 cells were plated in 96‐well plates at a density of 0.2 × 10^5^ cells per well and incubated overnight. After attachment, the cells were treated with increasing concentrations of nanoparticles (0, 10, 50, 100, and 200 µg/mL) for 24 h. Cell viability was assessed using the MTT assay to quantify survival rates across different treatment conditions.

### 4.17 Apoptosis Kit

To analyze apoptosis, 4T1 cells were cultured in six‐well plates at a density of 0.3 × 10^6^ cells per well and allowed to attach overnight. The following day, cells were incubated with nanoparticles (200 µg/mL) for 24 h. After treatment, cells were collected, rinsed with PBS, and stained with Annexin V‐FITC and PI for 30 min. Apoptotic populations were quantified using flow cytometry.

### 4.18 Thiol Tracker

To assess intracellular glutathione (GSH) levels, 4T1 cells were cultured on eight‐well confocal slides (0.2 × 10^5^ cells per well) and allowed to attach overnight. After treating cells with nanoparticles (20 µg/mL) for 4 h, they were stained with thiol tracker violet for 20 min. Fluorescence signals were then acquired using confocal microscopy to evaluate GSH depletion.

### 4.19 DCFH‐DA (ROS) Assay

For intracellular ROS detection, 4T1 cells were seeded in eight‐well chambered slides (0.2 × 10^5^ cells per well) and left overnight to adhere. Cells were treated with nanoparticles (20 µg/mL) for 4 h, then incubated with DCFH‐DA for 20 min. After washing with PBS, nuclear staining was performed using Hoechst 33342 (1 µg/mL). Confocal microscopy was used to capture the fluorescence intensity and assess ROS production.

For the Flow Cytometry DCFH‐DA ROS Assay, 4T1 cells were seeded in six‐well plates (2 × 10^5^ cells/well) and treated with Control, PMC, or HA‐PGMC (20 µg/mL) for 4 h. Cells were then incubated with DCFH‐DA (10 µm) for 20 min, washed twice with PBS, harvested by trypsinization, and analyzed on a BD LSRFortessa flow cytometer (FITC channel).

### 4.20 Lipid Peroxidation (LPO) Assay

4T1 cells were seeded in eight‐well chambered slides at a density of 0.2 × 10^5^ cells per well and incubated overnight for cell adherence. The following day, cells were exposed to nanoparticles (20 µg/mL) for 4 h. Post‐treatment, LPO‐specific fluorescent dye was applied for 20 min to assess lipid peroxidation levels. After triple PBS rinses, nuclei were counterstained with Hoechst 33342 (1 µg/mL) for 10 min. Fluorescence imaging was carried out using a confocal laser scanning microscope.

### 4.21 JC‐1 Mitochondrial Membrane Potential Assay

To evaluate mitochondrial membrane potential, 4T1 cells were cultured on eight‐well confocal slides at a seeding density of 0.2 × 10^5^ cells per well and left overnight. The next day, cells were treated with nanoparticles (20 µg/mL) for 4 h. JC‐1 working solution was then added and incubated for 30 min. Following three washes with JC‐1 staining buffer, fluorescence images were acquired via confocal microscopy.

### 4.22 HMGB1 and CRT Immunofluorescence

4T1 cells were plated onto eight‐well chambered coverslips at a density of 0.2 × 10^5^ cells per well and treated with different nanoparticle formulations (200 µg/mL) for 24 h. After treatment, cells were fixed, permeabilized, and incubated overnight with primary antibodies targeting HMGB1 and calreticulin (CRT). The next day, excess antibody was removed with PBS washes, and nuclei were counterstained with DAPI for 10 min. Confocal microscopy was used to visualize fluorescence and assess protein expression.

### 4.23 ATP Release Assay

To quantify ATP secretion, 4T1 cells were seeded in 96‐well plates (0.2 × 10^5^ cells/well) and treated with nanoparticles (200 µg/mL) for 24 h. After incubation, supernatants were collected, and ATP levels were measured according to the instructions provided by the commercial assay kit.

### 4.24 Hemolysis Assay

To evaluate hemocompatibility, a hemolysis assay was performed using fresh red blood cells (RBCs) isolated from the whole blood of healthy BALB/c mice. Briefly, blood samples were centrifuged at 4000 rpm for 10 min, and RBCs were washed five times with PBS until the supernatant was clear. Nanoparticles (NPs) were dispersed in PBS at final concentrations of 0, 5, 10, 20, 50, 100, 200, 400, and 800 µg/mL (200 µL total volume). PBS and deionized water were used as negative and positive controls, respectively. RBC suspensions (10 µL) were added to each NP solution, followed by incubation at room temperature for 2 h. The samples were then centrifuged at 4000 rpm for 5 min, and the supernatants were collected. Hemoglobin release was quantified by measuring absorbance at 570 nm using a microplate reader (Multiskan FC, Thermo Fisher Scientific, USA). The hemolysis percentage was calculated according to the following formula:

$$\begin{array}{c} \mathrm{Hemolysis}\;\left(\%\right)=\frac{\mathrm{Absorbanc}e_{\mathrm{sample}}-\mathrm{Absorbanc}e_{\mathrm{negative}\;\mathrm{control}}}{\mathrm{Absorbanc}e_{\mathrm{positive}\;\mathrm{control}}-\mathrm{Absorbanc}e_{\mathrm{negative}\;\mathrm{control}}}\\ \times 100\% \end{array}$$

### 4.25 Western Blot Analysis

Whole‐cell lysates were prepared in RIPA buffer (Epizyme, Cat. #PC101) with protease/phosphatase inhibitor cocktail (Epizyme, Cat. #GRF103). Protein concentrations were measured by BCA assay (Thermo Fisher Scientific, Cat. #23225). Equal amounts of protein (20 µg) were denatured in 5× SDS loading buffer (Epizyme, Cat. #LT103), separated on 10% SDS‐PAGE gels, and transferred to 0.45 µm PVDF membranes (Thermo Fisher Scientific, Cat. #88518). After blocking with 5% BSA in TBST (Epizyme, Cat. #TF103), membranes were probed with primary antibodies against CRT, HMGB1, and β‐actin (loading control) overnight at 4°C, followed by HRP‐conjugated secondary antibodies. Signals were detected using Pierce ECL substrate (Thermo Fisher Scientific, Cat. #32209) on a Bio‐Rad ChemiDoc system.

### 4.26 GOx Activity Assay

To evaluate whether encapsulation preserved GOx function, the catalytic activity of free GOx and HA‐PGMC‐encapsulated GOx was measured using a glucose‐consumption assay. Equivalent amounts of GOx (based on BCA quantification) were incubated with 500 µm glucose in PBS (pH 5.5) at 37 °C. Residual glucose was measured at defined time points using a glucose oxidase kit from beyotime. Relative GOx activity (%) was calculated as the ratio of the glucose consumption rate of encapsulated GOx to free GOx (set as 100%). Data are presented as mean ± SD (n = 4).

### 4.27 Hypoxic H_2_O_2_ Generation

To evaluate H_2_O_2_ generation under hypoxic conditions, 200 µL HA‐PGMC (1 mg/mL) was placed in N_2_‐saturated PBS (pH 5.5, 500 µm glucose) and sealed in a degassing chamber to maintain hypoxic conditions. H_2_O_2_ concentration was measured at defined time points using the same H_2_O_2_ kit as described above. Normoxic controls were run in parallel under ambient air.

### 4.28 In Vivo Studies

Animal experiments complied with all institutional and national requirements for the care and use of laboratory animals and were approved by the Animal Ethics Committee of the Chinese University of Hong Kong (25‐052‐MIS).

### 4.29 Tumor Model Establishment

Female BALB/c mice (4–6 weeks old) were used for all in vivo experiments. 4T1 murine breast cancer cells were cultured to approximately 80% confluency, harvested by trypsinisation, washed twice with sterile PBS, and resuspended at a concentration of 1 × 10^7^ cells/mL. For the single tumor model, 1 × 10^6^ cells in 100 µL PBS were injected subcutaneously into the right flank of each mouse [34]. Tumor dimensions were measured every two days using digital callipers, and tumor volume was calculated according to the formula: V = (length × width^2^) / 2. Treatment was initiated when tumor volumes reached approximately 60–80 mm^3^. For the bilateral tumor model, 1 × 10^6^ cells in 100 µL PBS were inoculated subcutaneously into the right flank (primary tumor) on Day −7, followed by inoculation of the same cell number into the left flank (distant tumor) on Day −4. Intratumoral treatment was administered to the primary tumor only, while the distant tumor remained untreated throughout the experiment to assess systemic antitumor immune activation (abscopal effect) [35, 36].

### 4.30 Biodistribution

Biodistribution of nanoparticles was evaluated using an IVIS spectrum imaging system. Female BALB/c mice (4–6 weeks old, n = 4) were subcutaneously inoculated with 4T1 cells (1 × 10^6^ cells in 100 µL PBS) into the flank to establish tumor xenografts. When tumors reached ∼100 mm^3^, mice were randomly assigned into two groups and administered DiR‐labeled nanoparticles (PMC or HA‐PGMC) via tail vein injection. Whole‐body fluorescence imaging was performed at predetermined time points (0, 4, 8, 12, and 24 h). After 24 h, mice were sacrificed, and tumors along with major organs were excised for ex vivo fluorescence imaging and quantitative analysis.

### 4.31 In Vivo Antitumor Efficacy

Female BALB/c mice (4–6 weeks old) bearing subcutaneous 4T1 tumors (∼60‐80 mm^3^, 7–10 days post‐inoculation) were randomly divided into four groups (n = 8 per group): (1) PBS, (2) αPD‐L1(100 µg/dose), (3) HA‐PGMC (mil‐100 equivalent 15 mg/kg), and (4) HA‐PGMC (mil‐100 equivalent 15 mg/kg) + αPD‐L1. Treatments were administered every 2 days for a total of four cycles (on Days 0, 2, 4, and 6 post‐initial‐treatment): nanoparticles via intravenous injection and αPD‐L1via intraperitoneal injection on the following day. Tumor volumes and body weights were monitored every other day, with tumor volume calculated as (width^2^ × length)/2. Mice were sacrificed on Day 14 post‐initial‐treatment (Day 21 post‐tumor‐inoculation), and blood was collected for biochemical analysis. Tumors and major organs were harvested, fixed in 4% paraformaldehyde, and processed for H&E and TUNEL staining to assess therapeutic efficacy.

### 4.32 Bilateral Tumor Model

To evaluate the systemic antitumor immune response (abscopal effect), a bilateral 4T1 tumor model was established. Female BALB/c mice (4–6 weeks old) were subcutaneously inoculated with 4T1 cells (1 × 10^6^ cells in 100 µL PBS) on the right flank (primary tumor, Day −7) and left flank (distant tumor, Day −4). When the primary tumor reached ∼60‐80 mm^3^, mice were randomly divided into four groups (n = 5 per group): (1) PBS, (2) αPD‐L1, (3) HA‐PGMC, and (4) HA‐PGMC + αPD‐L1. HA‐PGMC (mil‐100 equivalent 15 mg/kg) was administered via intratumoral (i.t.) injection into the primary tumor only, while αPD‐L1 (100 µg/dose) was administered intraperitoneally on the following day. Treatments were repeated every 2 days from Day 0 to Day 7. Mice were sacrificed on Day 12 post‐treatment (Day 19 post‐inoculation). Both primary and distant tumors were harvested, photographed, and weighed to assess local therapeutic efficacy and the abscopal immune response, respectively.

### 4.33 In Vivo Immune Evaluation

To evaluate immune activation, tumors were harvested on day 14 post‐treatment. Portions were fixed in 4% paraformaldehyde, sectioned, and subjected to immunofluorescence staining with anti‐CD206‐PE, anti‐CD86‐APC, and DAPI.

For flow cytometry analysis, tumors were minced into 2–4 mm fragments and digested in an enzyme mixture of collagenase IV (1 mg/mL), hyaluronidase (0.1 mg/mL), and DNase I (100 U/mL) at 37°C for 45 min. Cell suspensions were filtered, washed with cold PBS, and stained with fluorophore‐conjugated antibodies (APC‐CD4, PE‐CD8, APC‐CD80, PE‐CD86, FITC‐CD11b, APC‐CD206). Flow cytometry was used to quantify T cell subsets, dendritic cells, and macrophage polarization.

For tumor‐infiltrating regulatory T cell (Treg) analysis, single‐cell suspensions were surface‐stained with anti‐mouse CD45‐APC/Cy7 and CD4‐FITC antibodies for 30 min at 4 °C, followed by fixation and permeabilization using the FoxP3/Transcription Factor Staining Buffer Set (eBioscience, Cat. #00‐5523‐00) according to the manufacturer's protocol. Intracellular staining was performed with anti‐mouse FoxP3‐PE antibody. Tregs were identified as CD45^+^CD4^+^FoxP3^+^ cells and quantified as a percentage of the live CD45^+^ leukocyte population.

### 4.34 Biosafety

Biosafety was assessed after 14 days of treatment. Major organs (heart, liver, spleen, lung, and kidney) and blood samples were collected. Organs were weighed, fixed in 4% paraformaldehyde, and sectioned for histological evaluation by H&E staining to assess potential tissue abnormalities. Serum was isolated for blood biochemical analysis, including alanine aminotransferase (ALT), aspartate aminotransferase (AST), creatine kinase (CK), creatinine (CRE), and blood urea nitrogen (BUN), to evaluate hepatic, renal, and systemic safety.

### 4.35 ICP‐OES Biodistribution

For quantitative elemental biodistribution, major organs (heart, liver, spleen, lung, kidney) and tumor tissue were harvested at 24 h post‐injection from mice treated with PMC or HA‐PGMC (n = 5). Tissues were weighed, digested in 2 mL concentrated HNO_3_ overnight at 110°C, followed by the addition of 0.5 mL H_2_O_2_ to complete digestion. Samples were diluted with 2% HNO_3_ and analyzed by ICP‐OES for Cu and Fe content. Results are expressed as ppm (µg metal per g tissue).

### 4.36 Statistical Analysis

All quantitative data are presented as mean ± standard deviation (SD). No data pre‐processing, transformation, or outlier exclusion was applied. Sample sizes were n = 5 for all in vitro experiments, n = 4 for in vivo biodistribution studies, and n = 5 for in vivo antitumor efficacy experiments, unless otherwise stated. Data normality was assessed using the Shapiro‐Wilk test. For multi‐group comparisons, statistical significance was determined by one‐way ANOVA followed by Tukey's post‐hoc test when data were normally distributed, or by the non‐parametric Kruskal‐Wallis test followed by Dunn's post‐hoc test when normality was not met. For two‐group comparisons, an unpaired two‐tailed Student's t‐test or Mann‐Whitney U test was applied based on the same normality criterion. A *p*‐value <0.05 was considered statistically significant (^*^ *p* <0.05, ^**^ *p* <0.01, ^***^ *p* <0.001). All statistical analyses and data visualizations were performed using GraphPad Prism (version 9.0, GraphPad Software, San Diego, CA, USA) and OriginPro (version 2021, OriginLab Corporation, Northampton, MA, USA).

## Funding

This research was funded by the Hong Kong Research Grant Council and the Chinese University of Hong Kong.

## Conflicts of Interest

The authors declare no conflicts of interest.

## Supporting information




**Supporting File**: advs75465‐sup‐0001‐SuppMat.docx.

## Data Availability

The data that support the findings of this study are available in the supplementary material of this article.
