# Supplementary material for: ROS Self‐Supply Nanoplatform Based on Fenton Catalyst for Chemodynamic and Immunotherapy: Reprogramming Cold Tumor Into Hot Tumor in Cancer Treatment
Source: Adv Sci (Weinh). 2026 Apr 27:e23039. Online ahead of print. doi: 10.1002/advs.202523039 (PMC13335509; doi:10.1002/advs.202523039)
Supplement: Supplementary file 1 — Supporting File: advs75465‐sup‐0001‐SuppMat.docx. [file ADVS-9999-e23039-s001.docx]

Supporting Information

ROS Self-Supply Nanoplatform Based on Fenton Catalyst for Chemodynamic and Immunotherapy: Reprogramming Cold Tumor into Hot Tumor in Cancer Treatment

Man Lung Lee, Jack Chun Hin Chen, Wai Wing Cheng, Kwan Yee Lau, Hiu Yee Kwan, Ngar-Yun Ellen Poon* and Hung Wing Li*


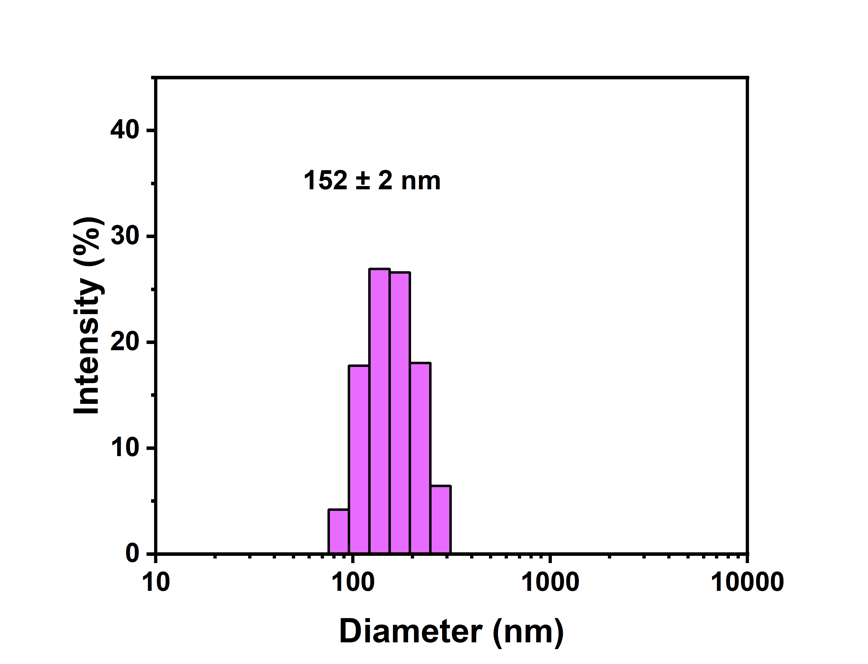


**Figure S1.** Average hydrodynamic size of mil-100 nanoparticles as measured by DLS.


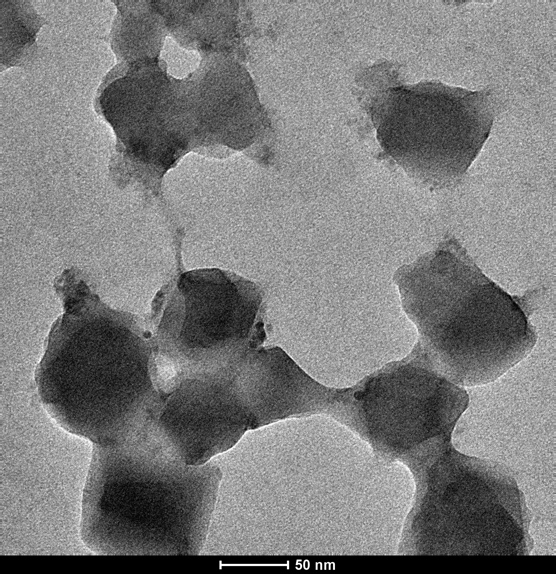


**Figure S2.** TEM image of MC.


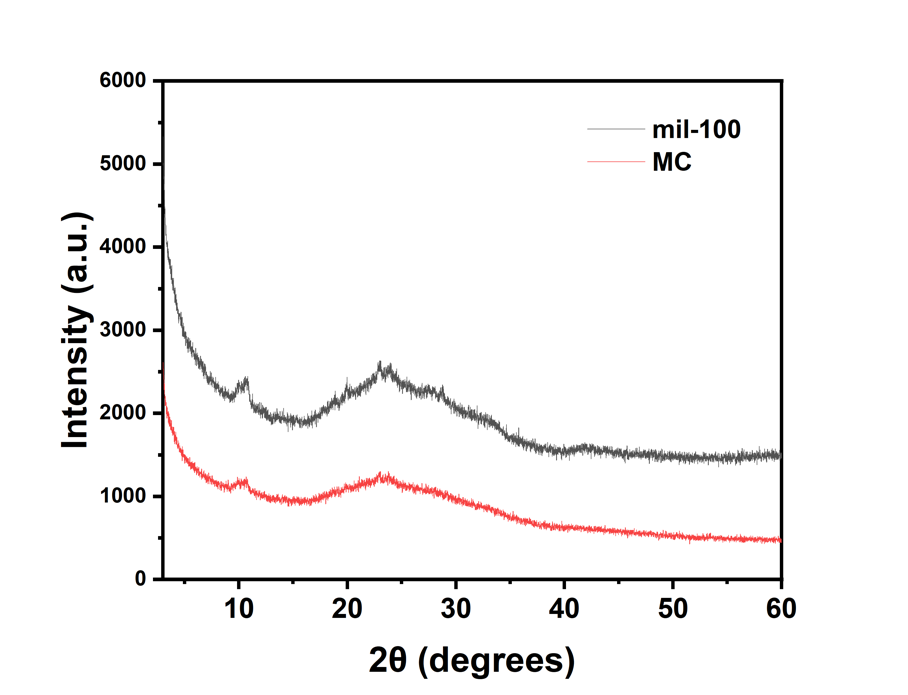


**Figure S3**. XRD spectrum of mil-100 and MC.

**Figure S4.** NMR spectrum of Peg_44_-PLA_106_

^1^H NMR (500 MHz, Chloroform-*d*) δ 5.25 – 5.11 (m, 212H), 3.65 (s, 176H), 3.38 (s, 3H), 1.62 – 1.51 (m, 636H).


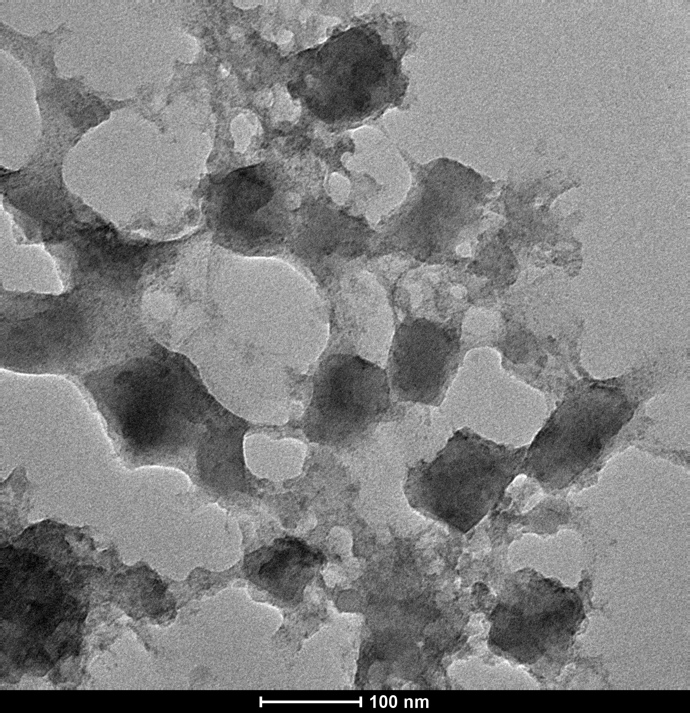


**Figure S5.** TEM image of PMC.


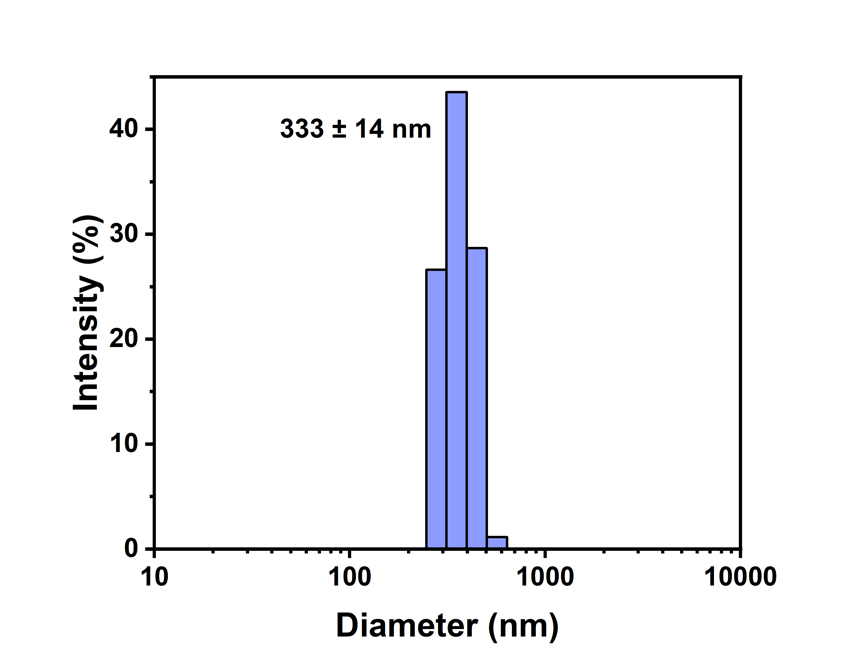


**Figure S6.** Average hydrodynamic size of PMC nanoparticles as measured by DLS.


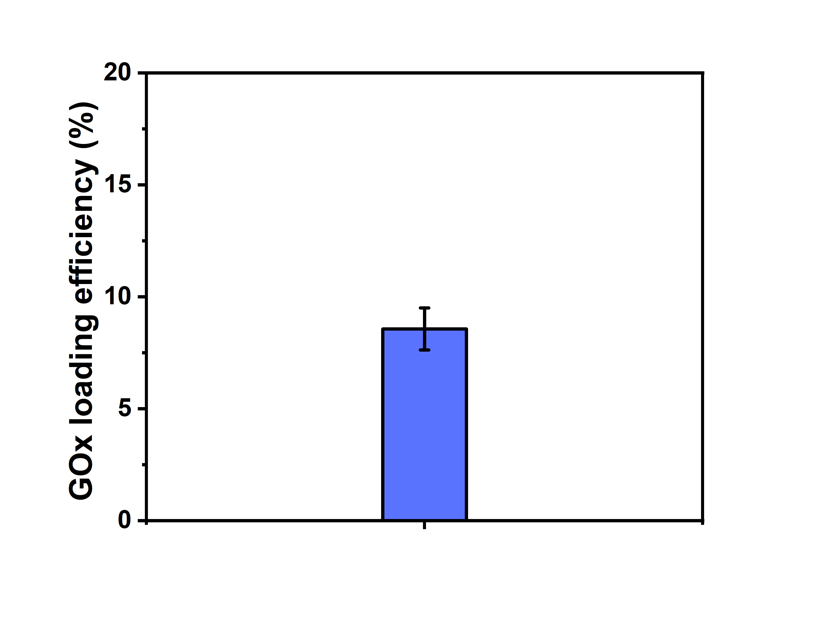


**Figure S7**. GOx loading efficiency in HA-PGMC.


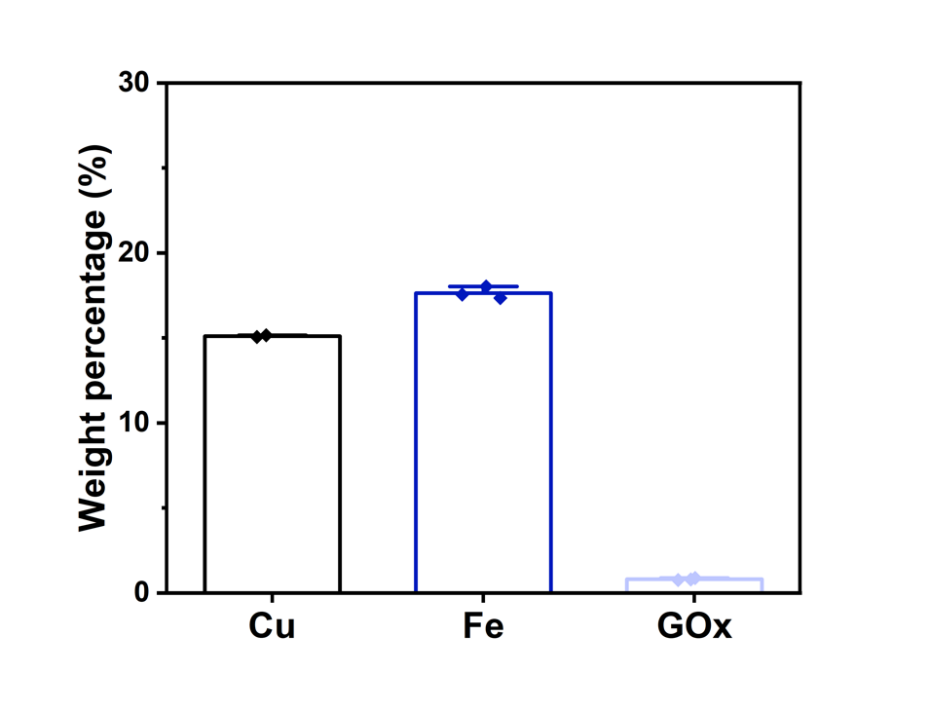


**Figure S8.** Weight percentage of Cu, Fe, and GOx in HA-PGMC. Cu and Fe contents were determined by ICP-OES, and GOx loading was quantified by BCA protein assay. Data are presented as mean ± SD (n = 3).


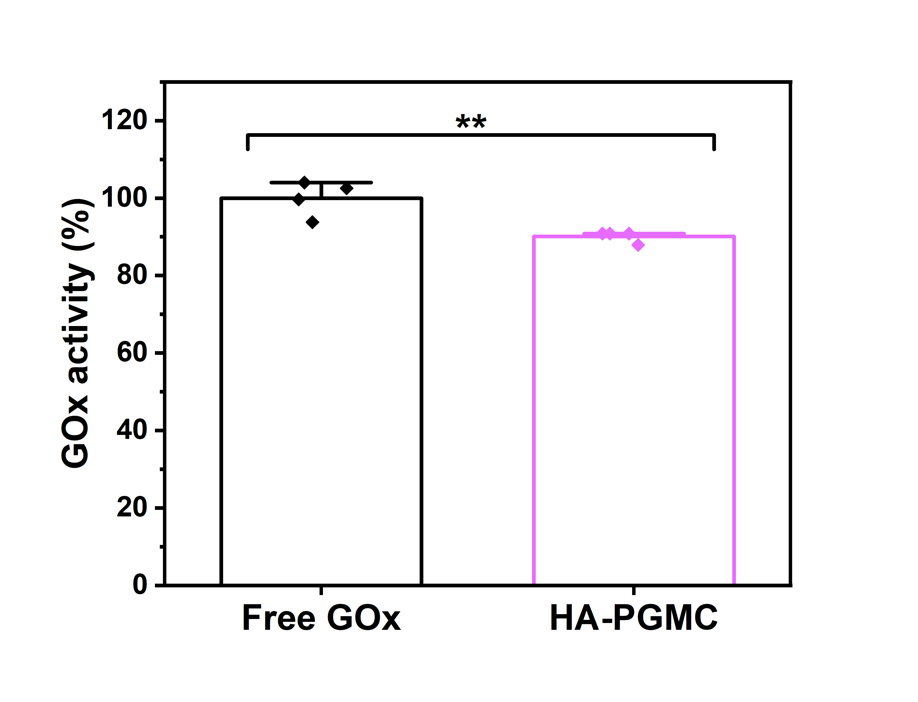


**Figure S9.** Catalytic activity of glucose oxidase (GOx) before and after encapsulation in HA-PGMC. GOx activity was evaluated by measuring glucose consumption and is presented as relative activity (%), normalized to free GOx (set as 100%). HA-PGMC retained approximately 90% of the enzymatic activity compared to free GOx. Data are expressed as mean ± SD (n = 4). Statistical significance was determined by one-way ANOVA.


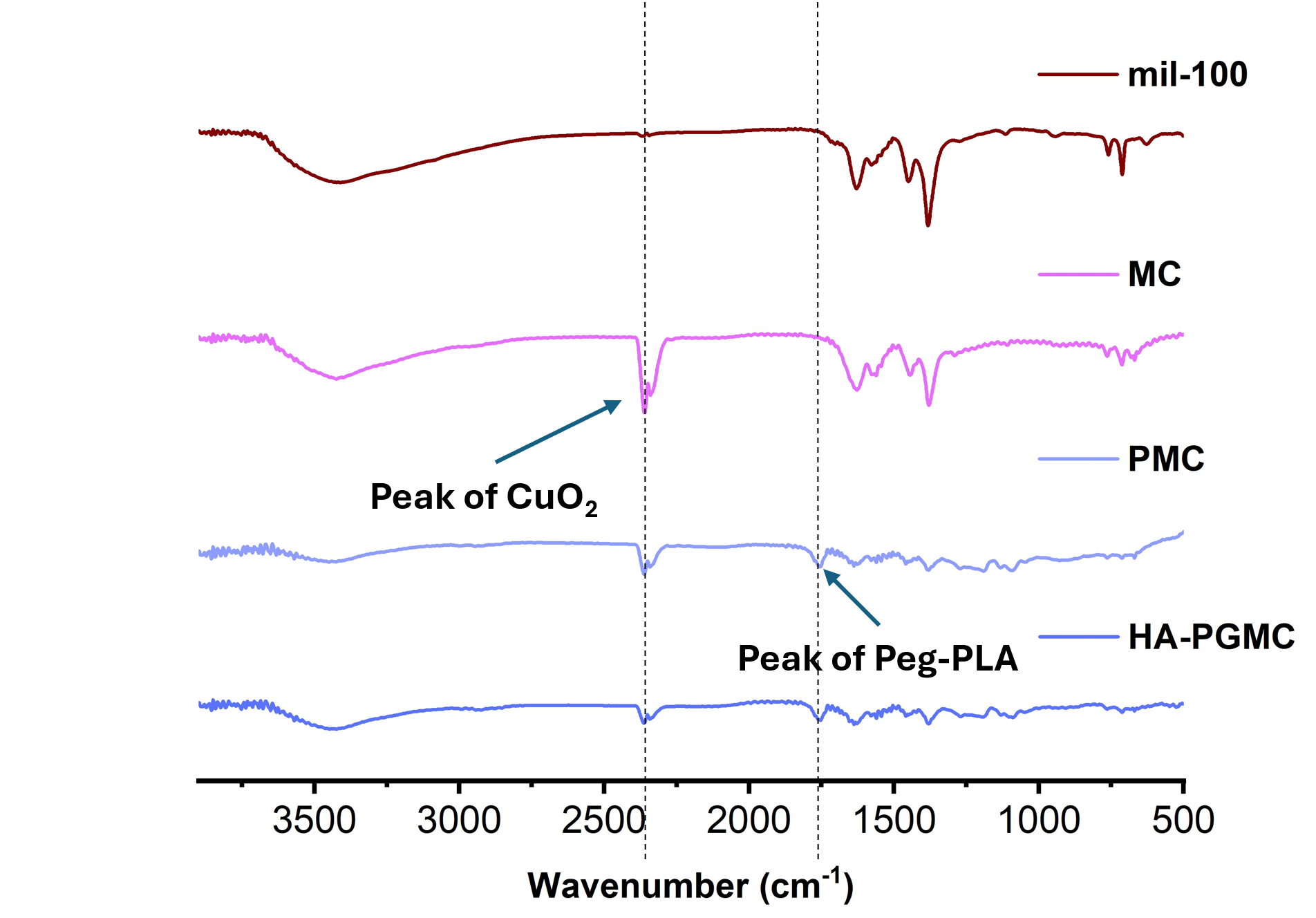


**Figure S10.** FTIR spectrum of mil-100, MC, PMC and HA-PGMC


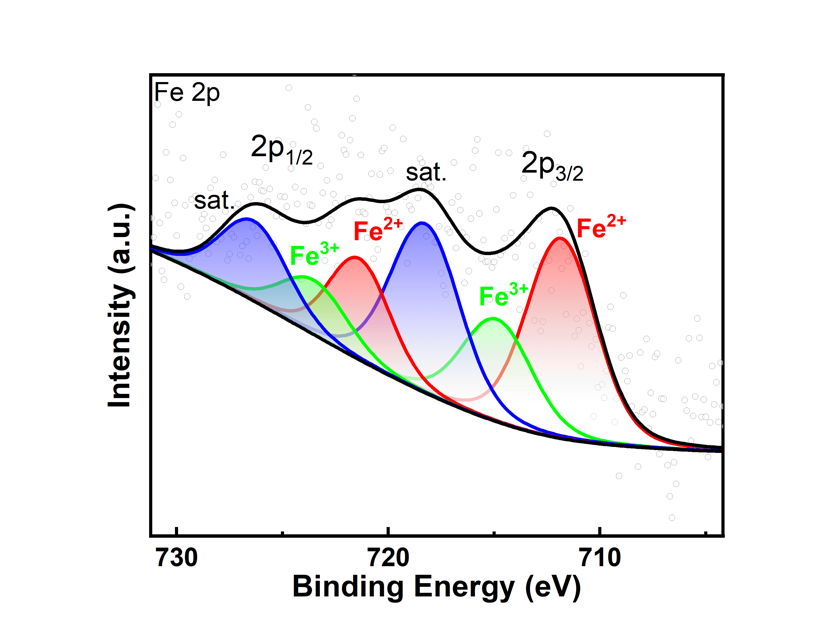


**Figure S11.** XPS spectrum of MC at Fe 2p.


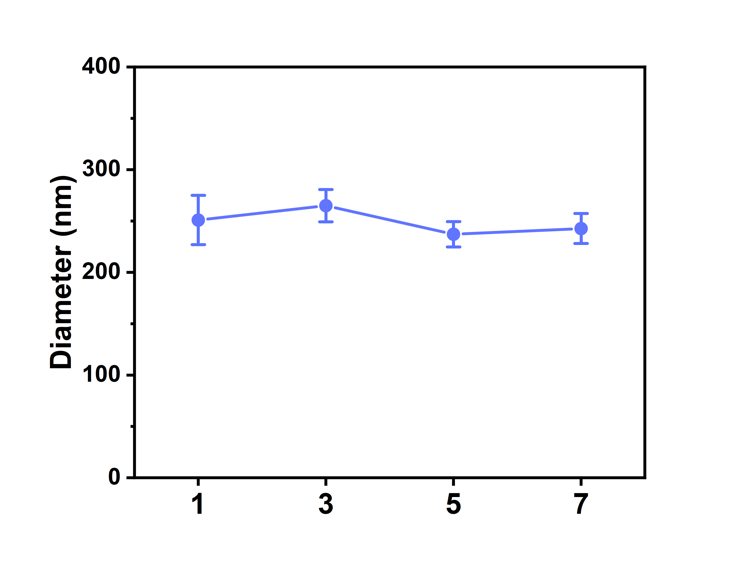


|  | **Day 1** | **Day 3** | **Day 5** | **Day 7** |
| --- | --- | --- | --- | --- |
| **Z-Ave (nm)** | 251.0 ± 9.7 | 265.0 ± 6.3 | 237.1 ± 5.0 | 242.6 ± 5.9 |
| **PDI** | 0.157 ± 0.050 | 0.175 ± 0.032 | 0.088 ± 0.054 | 0.143 ± 0.021 |

**Figure S12**. Hydrodynamic size and PDI of HA-PGMC in 7 days


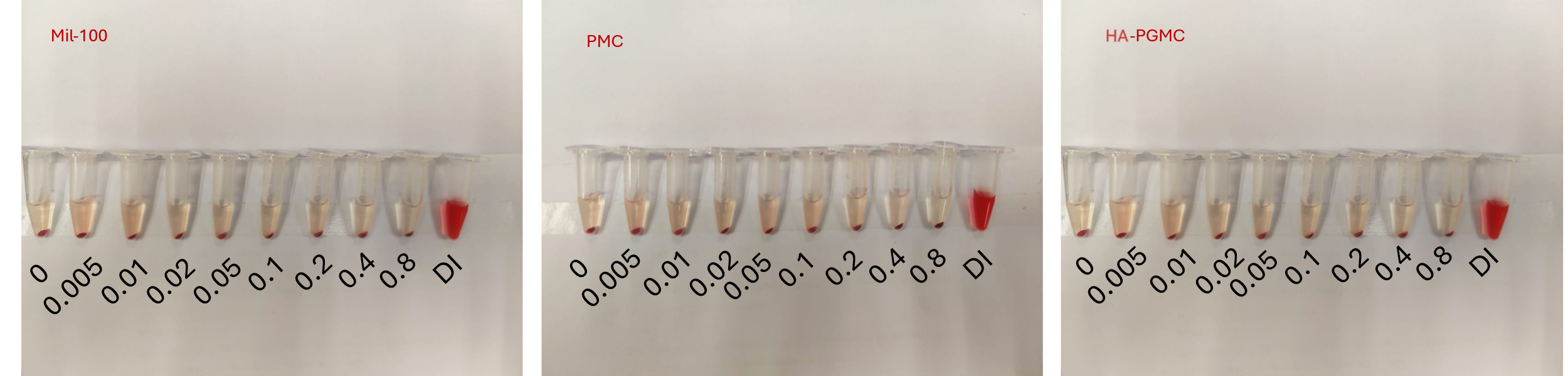


**Figure S13**. Hemolysis assay of different NPs in different concentrations (0-0.8 mg/mL).


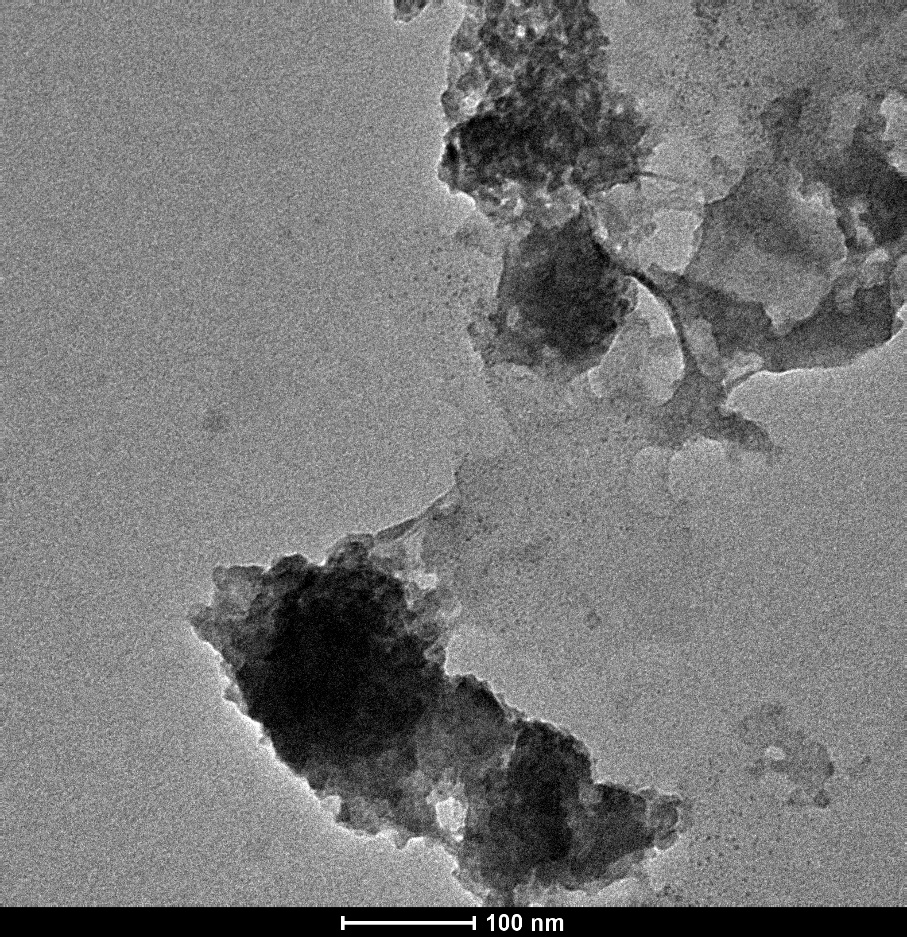

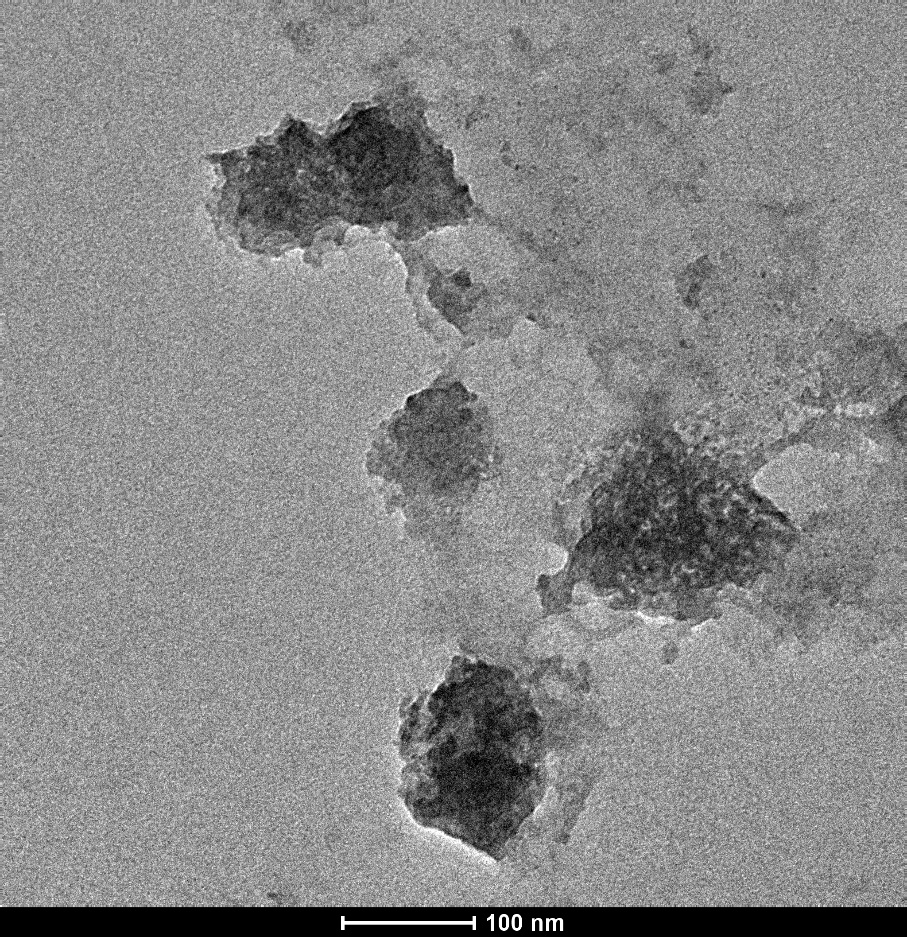

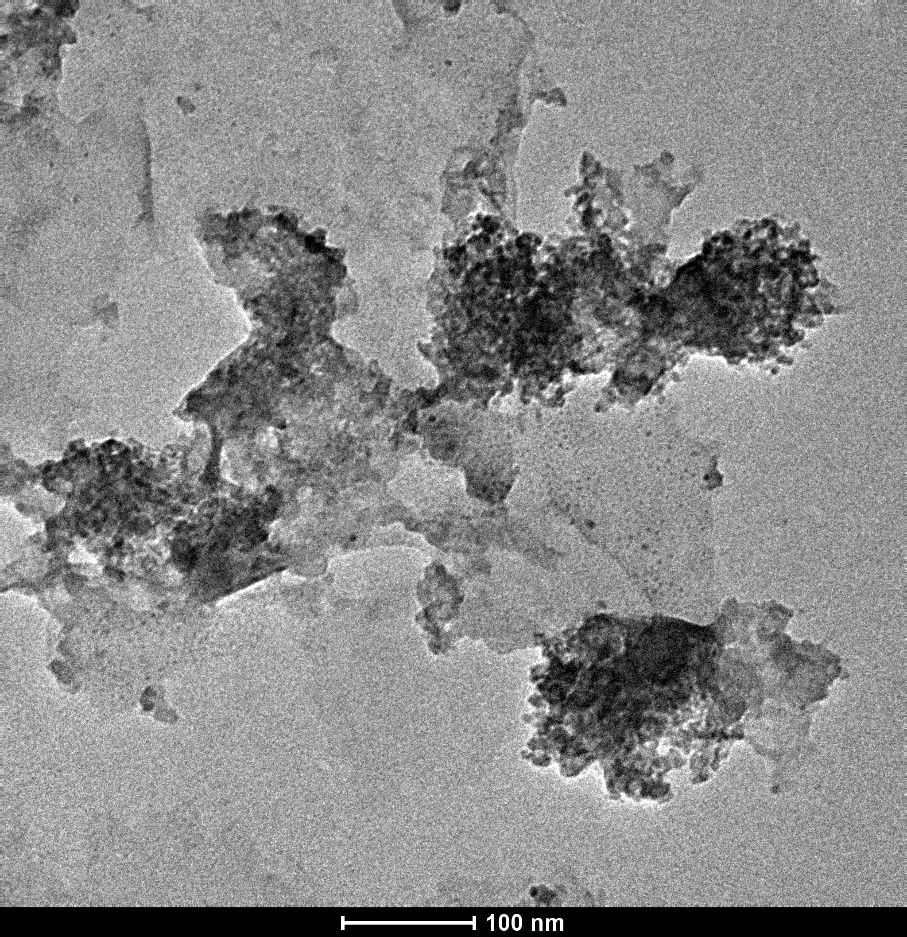

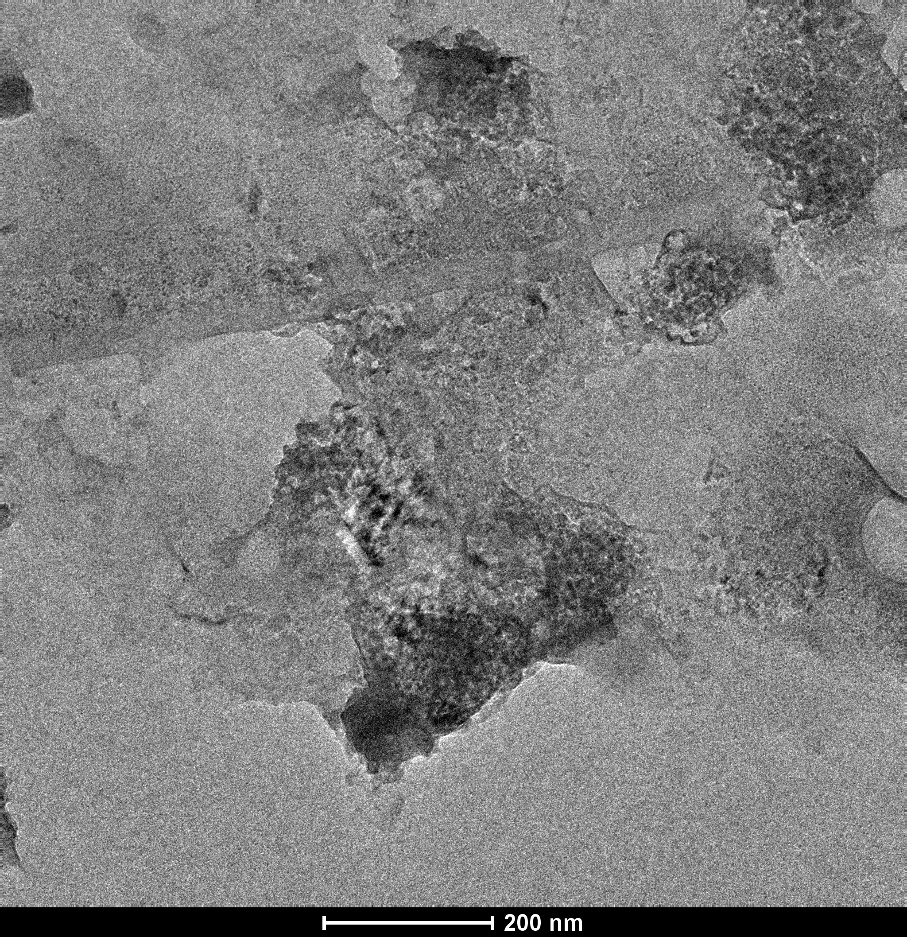


1 2 3 4

**Figure S14.** TEM images of HA-PGMC under different conditions incubated for 24 h: (1: 7.4, 0 µM glucose, 2: 7.4, 500 µM glucose, 3: 5.5, 0 µM glucose, 4: 5.5, 500 µM glucose).


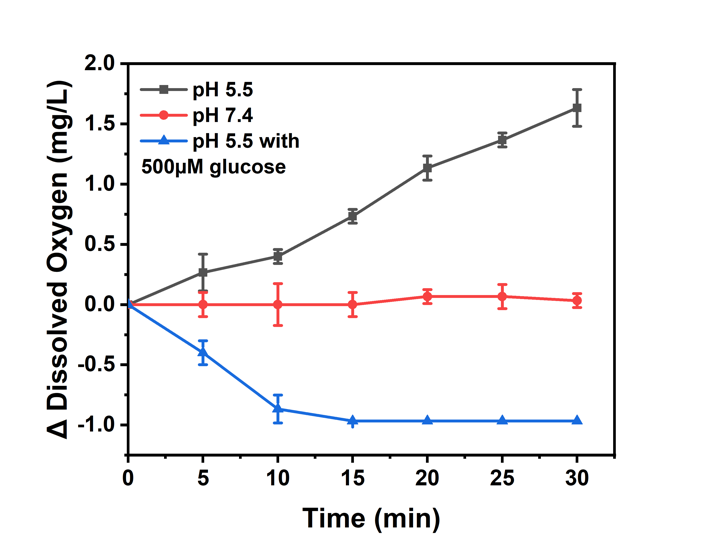


**Figure S15.** Dissolved oxygen of HA-PGMC in different pH and glucose levels.


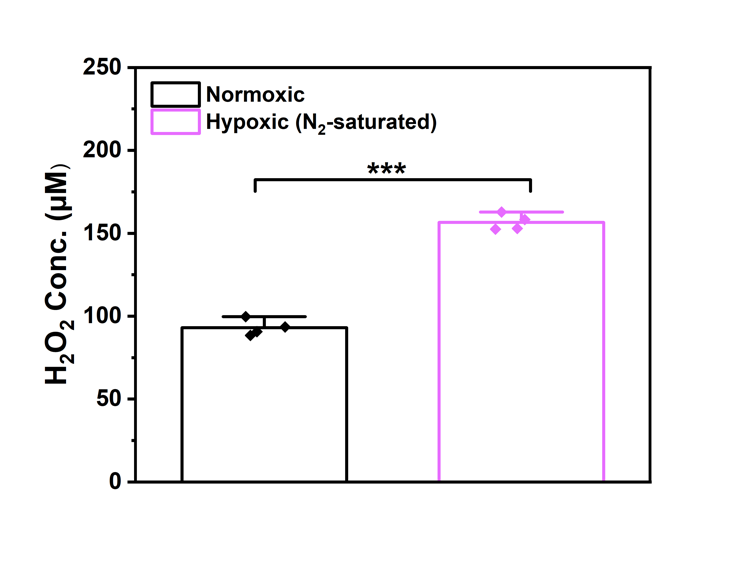


**Figure S16. H₂O₂ generation by HA-PGMC under normoxic and hypoxic conditions (N₂-**saturated)**.** Data are presented as mean ± SD (n = 3). Statistical significance was determined by one-way ANOVA (***p < 0.001).


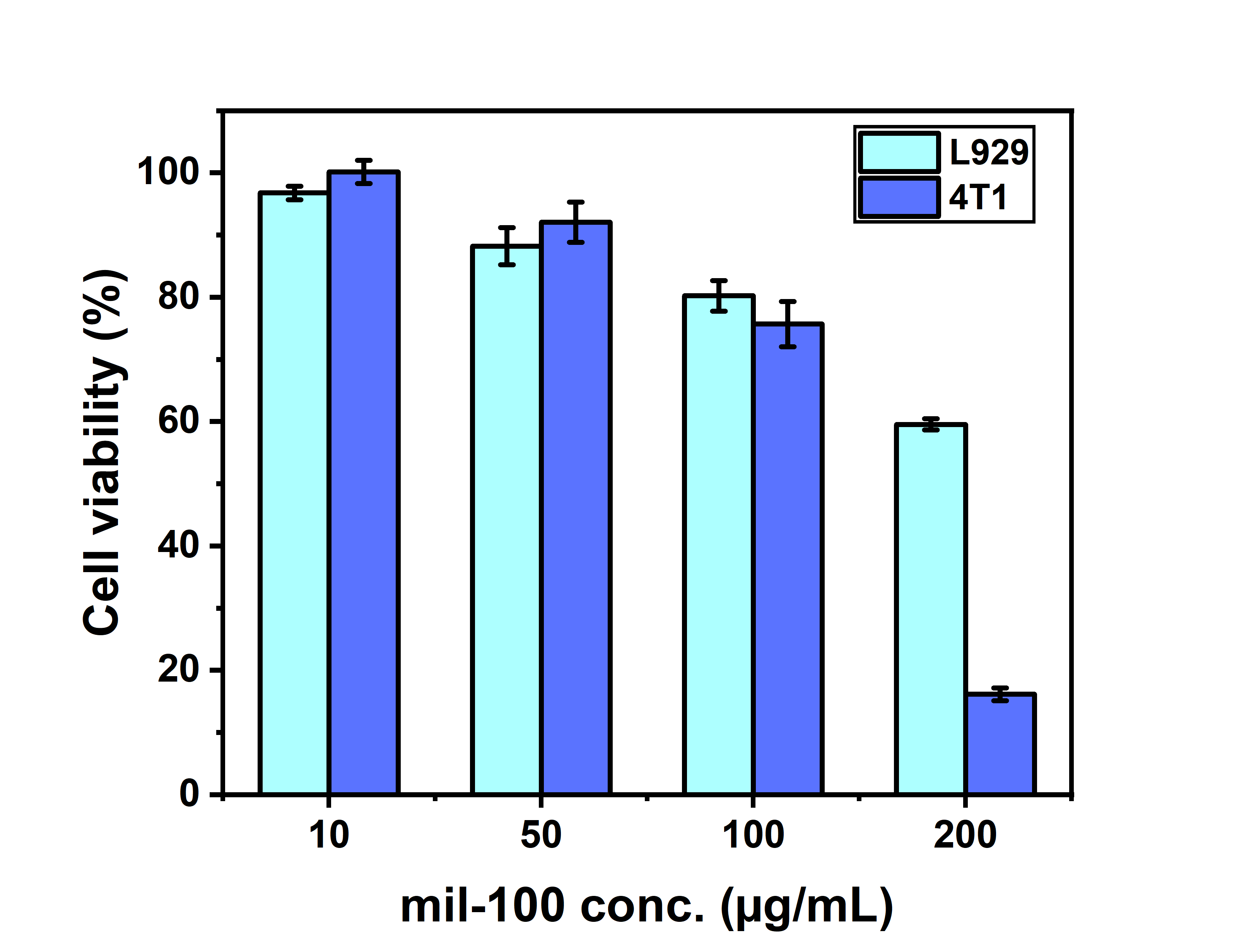


**Figure S17.** MTT assay of HA-PGMC in 4T1 cells and L929 cells


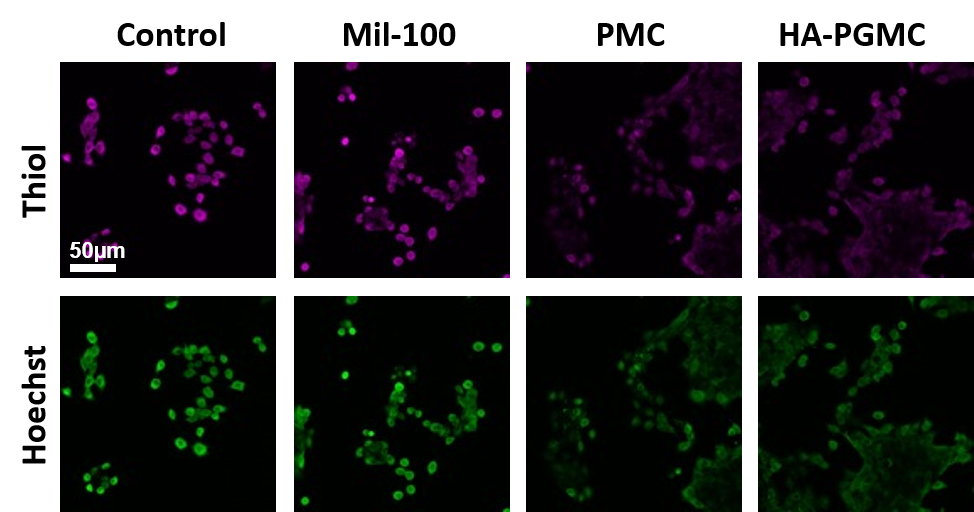


**Figure S18.** Thiol tracker with nuclear Hoechst staining scale bar: 100 μm.


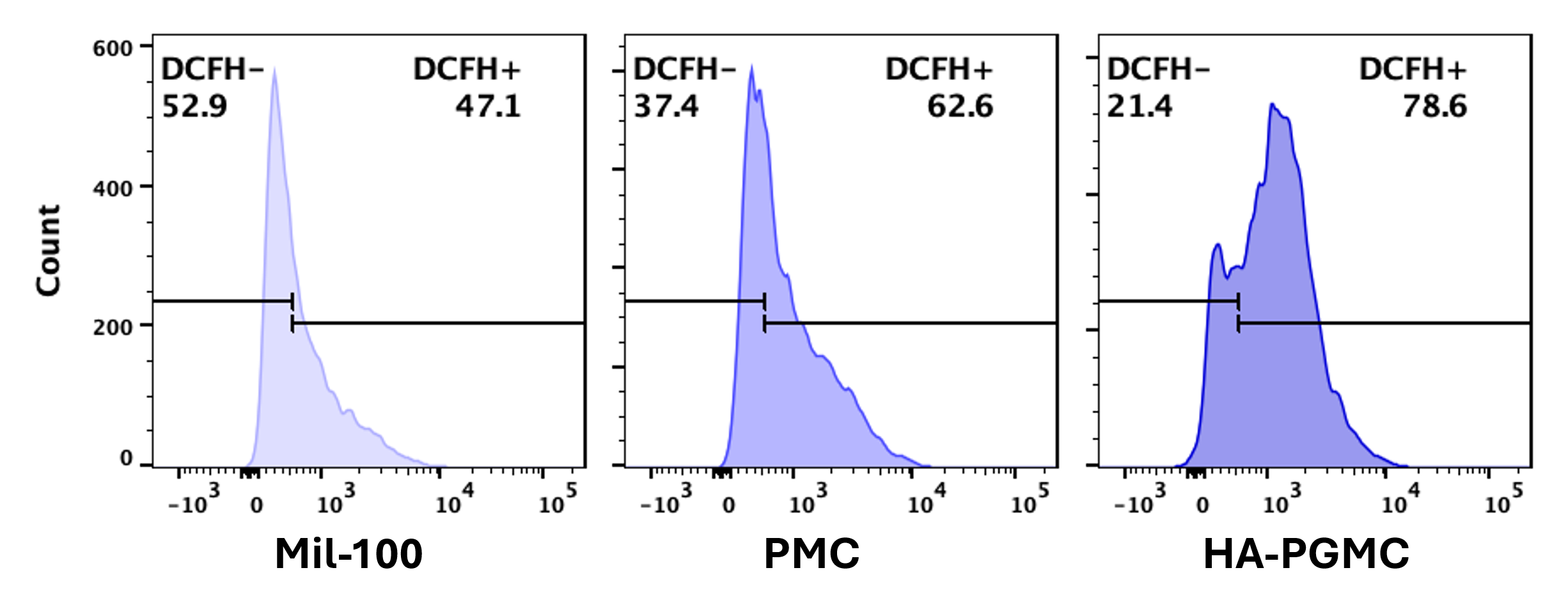


**Figure S19.** DCFH-DA flow cytometry analysis of intracellular ROS levels in 4T1 cells following treatment with Control, PMC, and HA-PGMC.


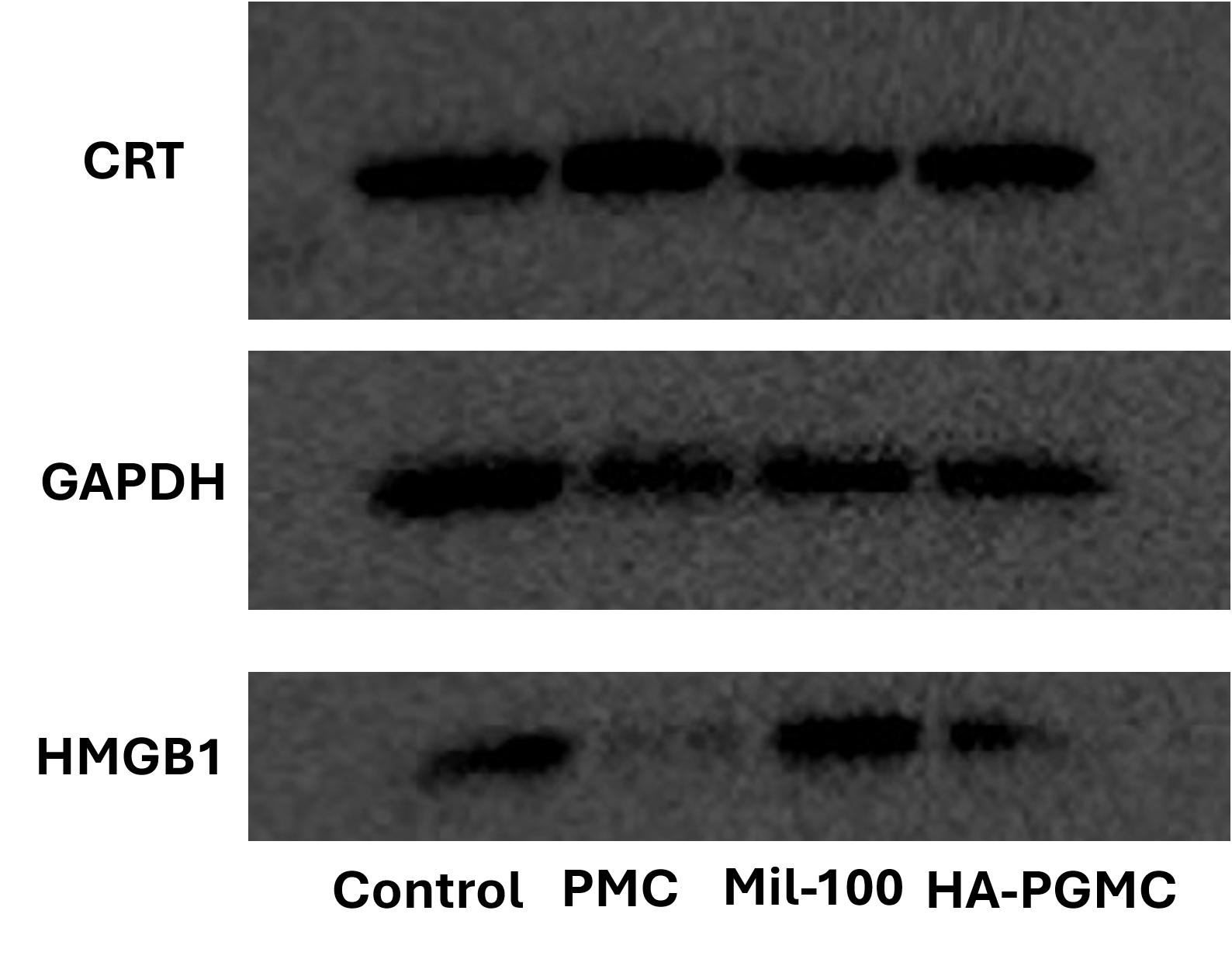


**Figure S20.** Western blot analysis of CRT and HMGB1 protein expression in 4T1 cells treated with Control, mil-100, PMC, and HA-PGMC.


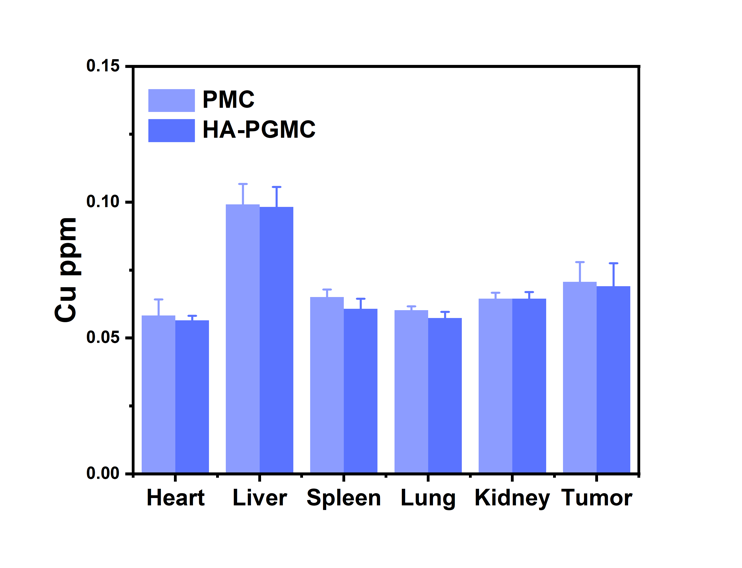


**Figure S21. Cu biodistribution in major organs and tumor measured by ICP-OES.** Cu concentration (ppm) in heart, liver, spleen, lung, kidney, and tumor of mice treated with PMC and HA-PGMC at 24 hours post-injection.

**
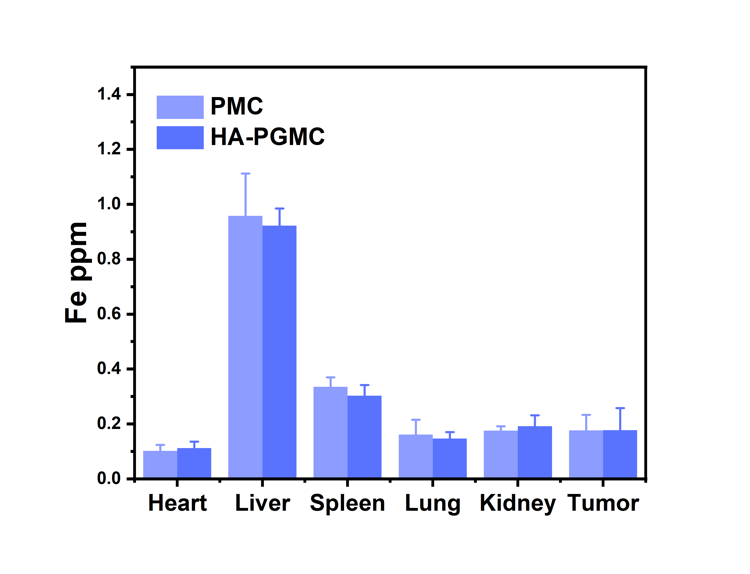
**

**Figure S22. Fe biodistribution in major organs and tumors measured by ICP-OES. F**e concentration (ppm) in heart, liver, spleen, lung, kidney, and tumor of mice treated with PMC and HA-PGMC at 24 hours post-injection.


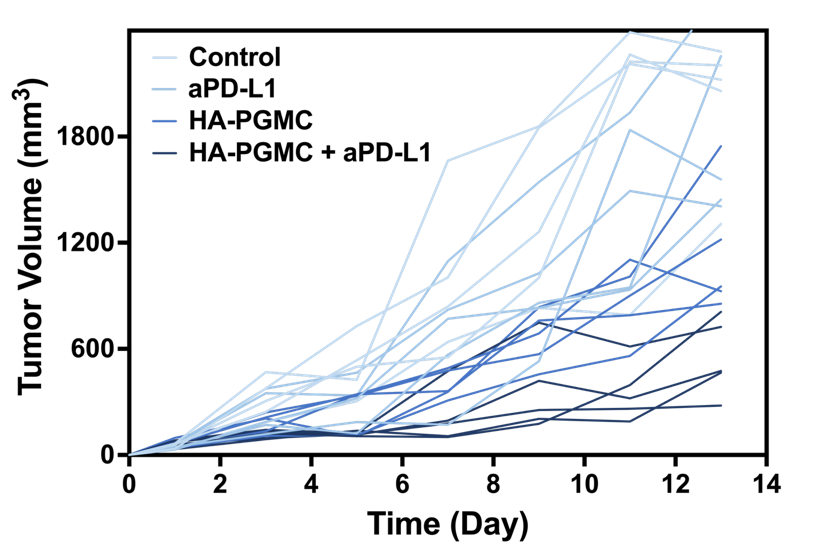


**Figure S23. Individual tumor volume growth curves for the single tumor model. Statistical analysis was performed using one-way ANOVA followed by Tukey’s post-hoc test for normally distributed data (verified by Shapiro-Wilk test), or Kruskal-Wallis test followed by Dunn's test for non-normally distributed data. (p < 0.05, **p < 0.01, **p < 0.001).**


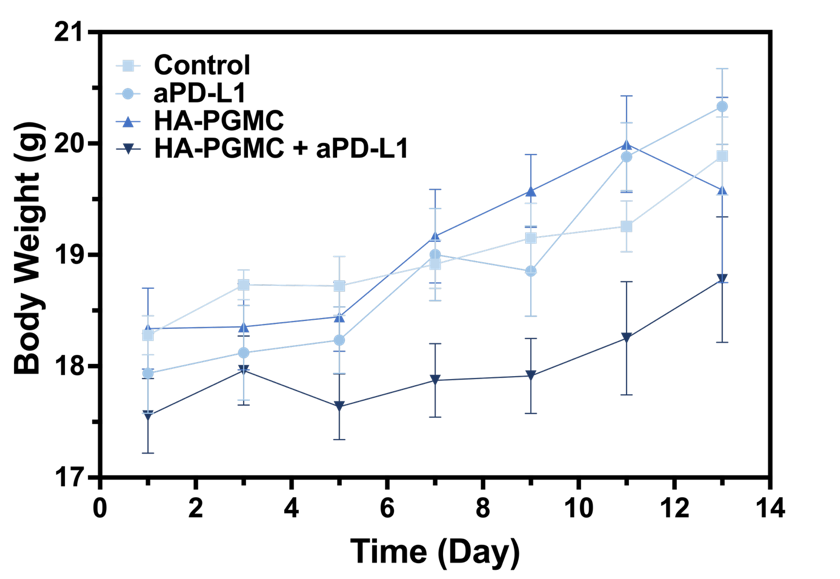


**Figure S24. Mean tumor volume growth curves for the single tumor model.** Mean tumor volume (mm³) ± SD of each treatment group over 14 days. Statistical analysis was performed using one-way ANOVA followed by Tukey’s post-hoc test for normally distributed data (verified by Shapiro-Wilk test), or Kruskal-Wallis test followed by Dunn's test for non-normally distributed data. (p < 0.05, **p < 0.01, **p < 0.001).


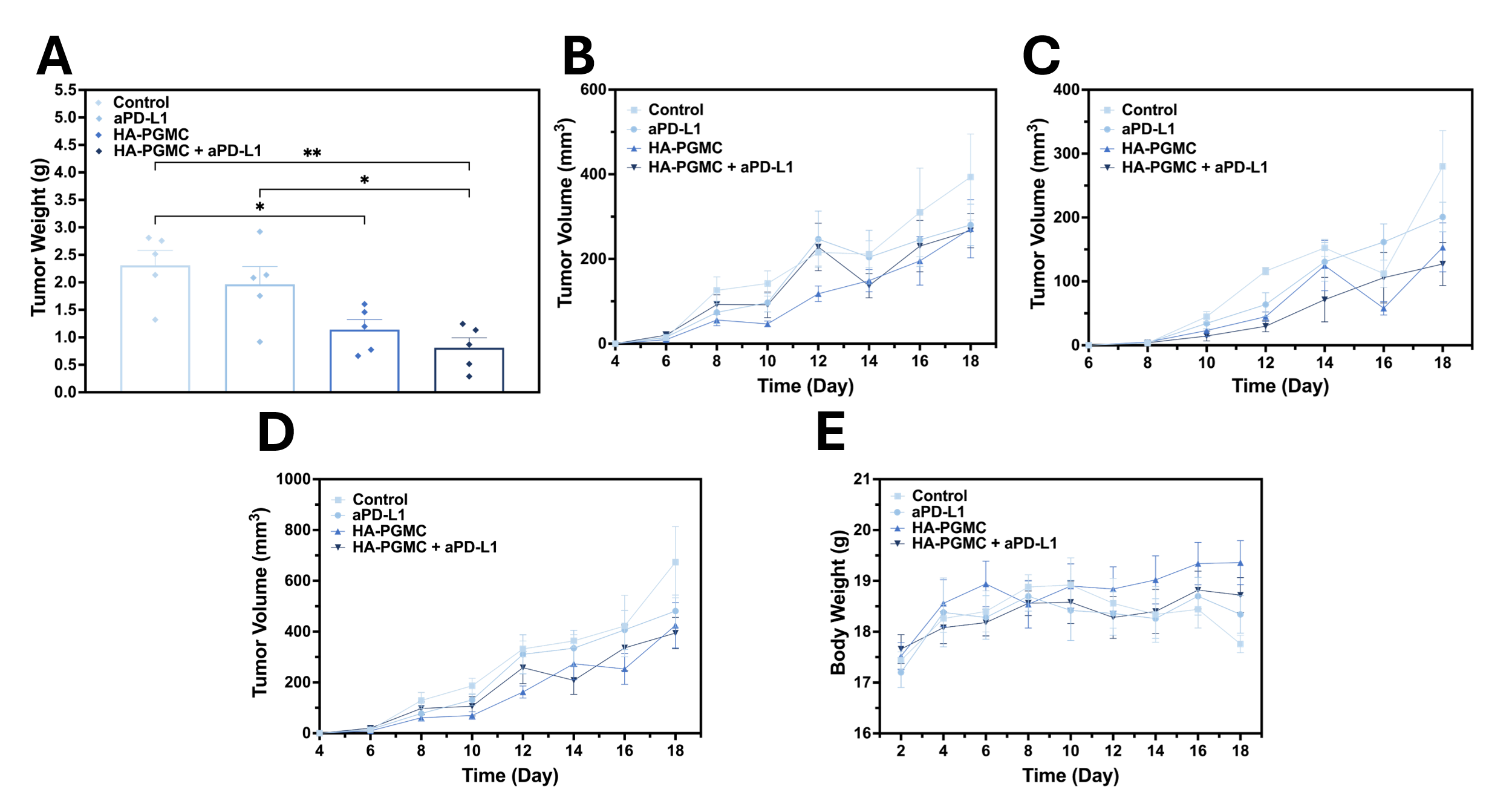


**Figure S25. In vivo antitumor efficacy of the bilateral tumor model.** (A) Total tumor weight of primary and distant tumors combined after 14 days of treatment. (B) Primary tumor volume growth curves over time. (C) Distant tumor volume growth curves over time. (D) Total tumor volume (primary + distant) growth curves over time. (E) Body weight of mice during treatment. Treatment groups: Control, αPD-L1, HA-PGMC, and HA-PGMC + αPD-L1 (n = 5 per group). Data are presented as mean ± SD. Statistical significance was determined by one-way ANOVA with Tukey's post-hoc test (*p < 0.05, **p < 0.01, ***p < 0.001).


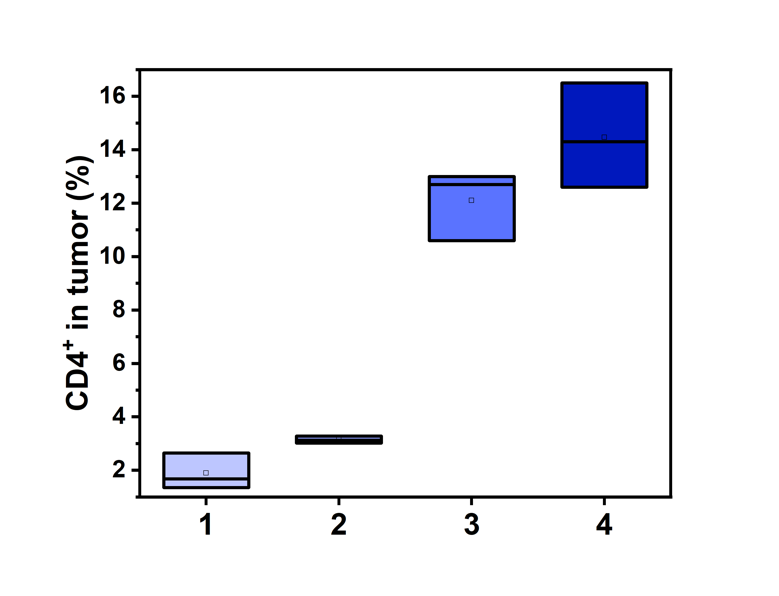


**Figure S26.** Flow cytometry analysis of CD4^+^ cell in tumor after different treatments (1: control, 2: αPD-L1, 3: HA-PGMC and 4: HA-PGMC + αPD-L1).


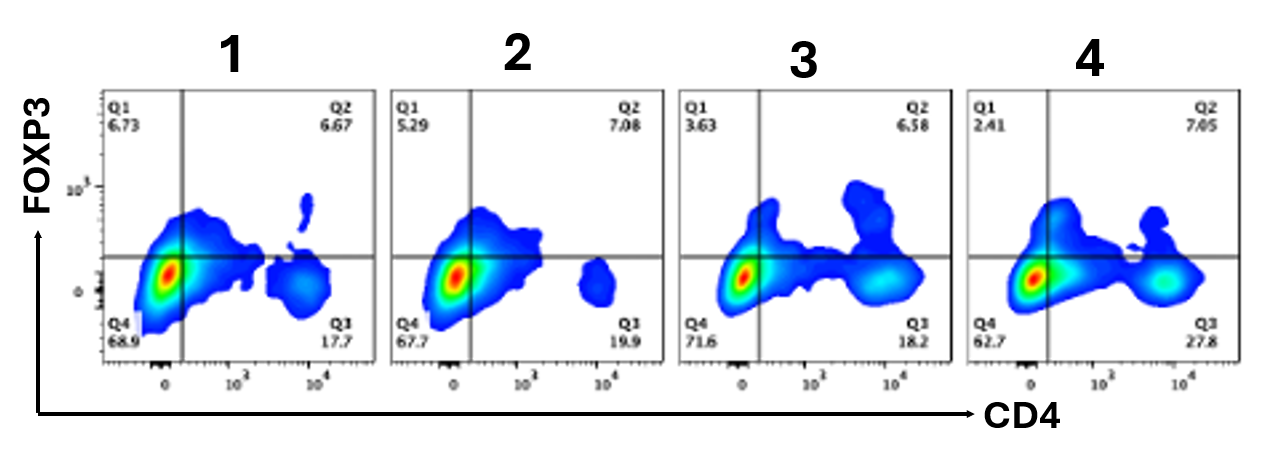


**Figure S27.** Representative flow cytometry dot plots of tumor-infiltrating regulatory T cells (Tregs) in 4T1 tumor-bearing mice after the indicated treatments. Cells were pre-gated on live CD45⁺ leukocytes and displayed as FoxP3 (y-axis) vs. CD4 (x-axis).


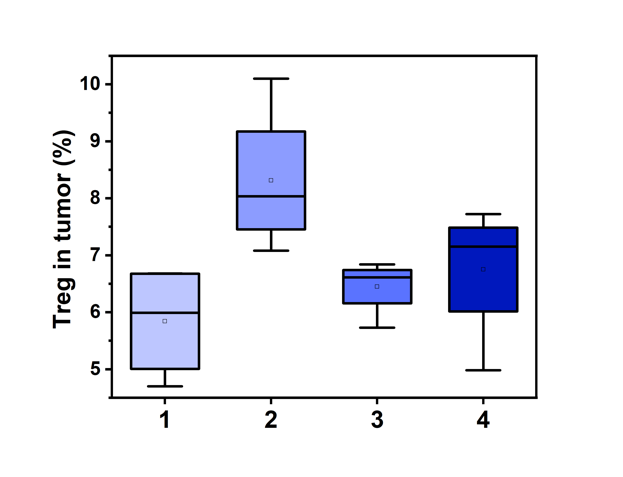


**Figure S28.** Quantification of tumor-infiltrating regulatory T cells (Tregs) across treatment groups. Treg frequency is expressed as the percentage of CD4⁺FoxP3⁺ cells within the live CD45⁺ leukocyte population isolated from 4T1 tumors. Groups: (1) Control, (2) αPD-L1, (3) HA-PGMC, and (4) HA-PGMC+αPD-L1 (n = 5)


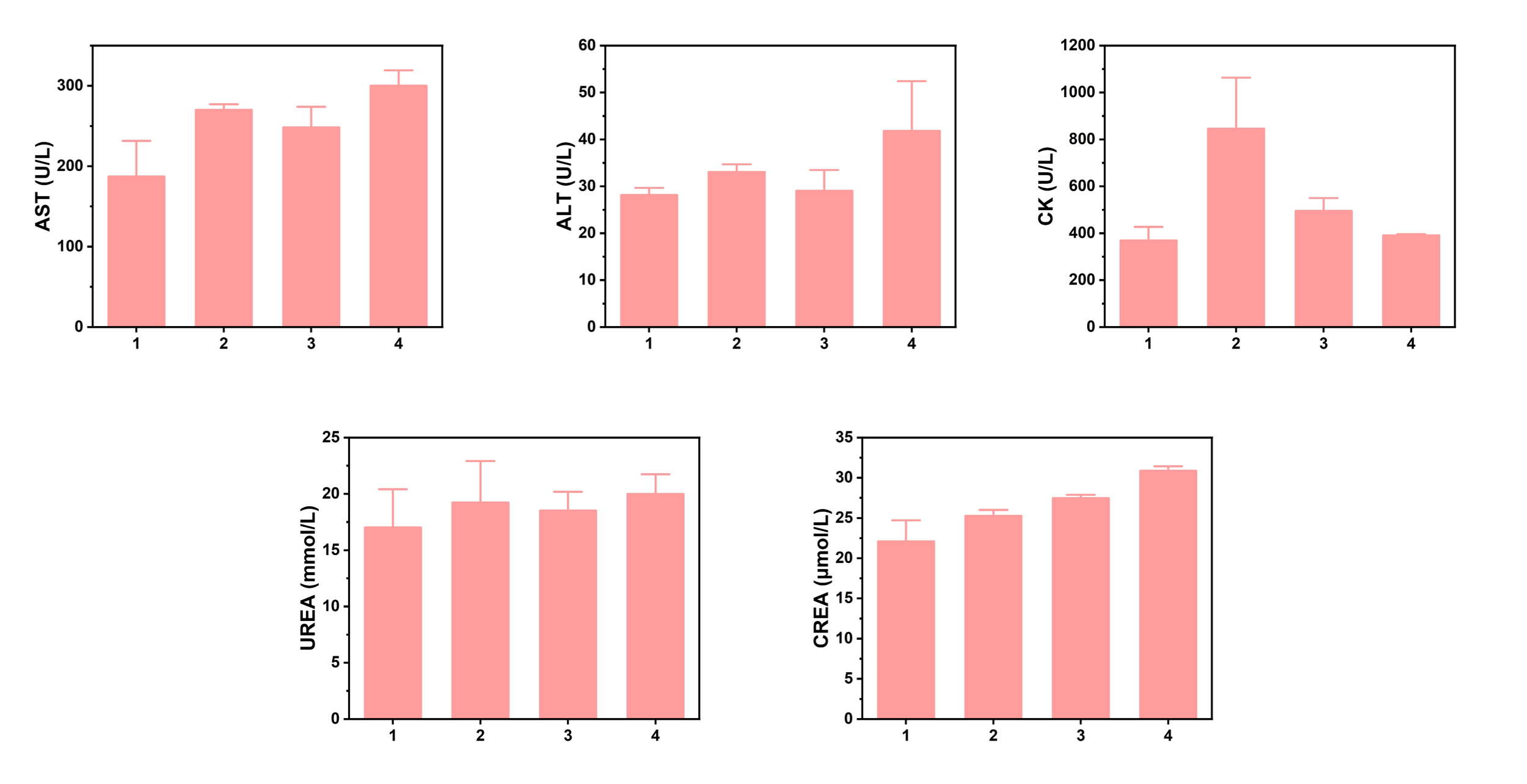


**Figure S29.** Blood biochemical analysis of mice after different treatments. Data are presented as mean ± SD (n = 3).


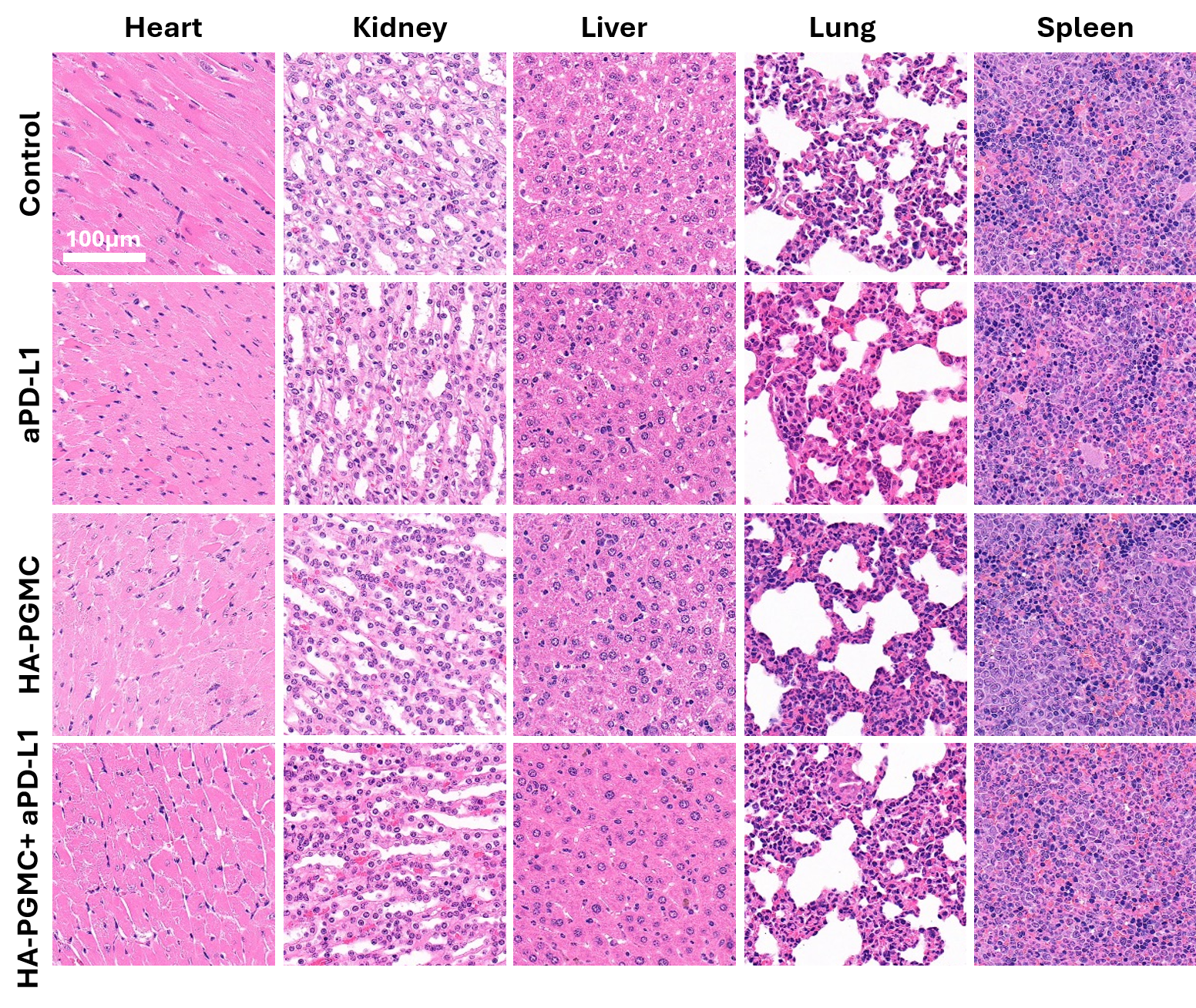


**Figure S30.** H&E staining of organs of different treatments (heart, kidney, liver, lung and spleen) scale bar: 100 μm.
